# Experimental approach and initial forest response to a simulated ice storm experiment in a northern hardwood forest

**DOI:** 10.1371/journal.pone.0239619

**Published:** 2020-09-25

**Authors:** Lindsey E. Rustad, John L. Campbell, Charles T. Driscoll, Timothy J. Fahey, Peter M. Groffman, Paul G. Schaberg, Gary J. Hawley, Ian Halm, Frank Bowles, Wendy Leuenberger, Geoffrey Schwaner, Gabriel Winant, Brendan Leonardi

**Affiliations:** 1 USDA Forest Service, Northern Research Station, Durham, New Hampshire, United States of America; 2 Department of Civil and Environmental Engineering, Syracuse University, Syracuse, New York, United States of America; 3 Department of Natural Resources, Cornell University, Ithaca, New York, United States of America; 4 Advanced Science Research Center at the Graduate Center, City University of New York, New York, New York, United States of America; 5 Cary Institute of Ecosystem Studies, Millbrook, New York, United States of America; 6 USDA Forest Service, Northern Research Station, Burlington, Vermont, United States of America; 7 Rubenstein School of Environment and Natural Resources, University of Vermont, Burlington, Vermont, United States of America; 8 USDA Forest Service, Northern Research Station, North Woodstock, New Hampshire, United States of America; 9 Research Designs, Lyme, New Hampshire, United States of America; 10 Department of Forestry and Natural Resources, University of Kentucky, Lexington, Kentucky, United States of America; 11 Hubbard Brook Research Foundation, North Woodstock, New Hampshire, United States of America; Trent University, CANADA

## Abstract

Ice storms are a type of extreme winter weather event common to north temperate and boreal forests worldwide. Recent climate modelling studies suggest that these storms may become more frequent and severe under a changing climate. Compared to other types of storm events, relatively little is known about the direct and indirect impacts of these storms on forests, as naturally occurring ice storms are inherently difficult to study. Here we describe a novel experimental approach used to create a suite of ice storms in a mature hardwood forest in New Hampshire, USA. The experiment included five ice storm intensities (0, 6.4, 12.7 and 19.1 mm radial ice accretion) applied in a single year, and one ice storm intensity (12.7 mm) applied in two consecutive years. Results demonstrate the feasibility of this approach for creating experimental ice storms, quantify the increase in fine and coarse woody debris mass and nutrients transferred from the forest canopy to the soil under the different icing conditions, and show an increase in the damage to the forest canopy with increasing icing that evolves over time. In this forest, little damage occurred below 6.4 mm radial ice accretion, moderate damage occurred with up to 12.7 mm of accretion, and significant branch breakage and canopy damage occurred with 19.1 mm of ice. The icing in consecutive years demonstrated an interactive effect of ice storm frequency and severity such that some branches damaged in the first year of icing appeared to remain in the canopy and then fall to the ground in the second year of icing. These results have implications for National Weather Service ice storm warning levels, as they provide a quantitative assessment of ice-load related inputs of forest debris that will be useful to municipalities creating response plans for current and future ice storms.

## Introduction

The increase in the frequency, intensity and severity of extreme weather events is emerging as a significant concern of climate change in the Anthropocene [1, 2]. These types of episodic events can have a greater impact on natural and managed ecosystems than the more gradual changes in mean temperature and precipitation that are typically associated with climate change [3, 4]. Despite the importance of these extreme weather events in shaping ecosystems, relatively little is known about their short- or long-term impacts. They are inherently difficult to study due to infrequent return intervals, high spatial variation in occurrence and intensity, difficulty to forecast reliably, and safety concerns [5, 6]. Much of the literature on ecosystem response to extreme weather events is derived from *post facto* analyses following naturally occurring events such as ice storms, late spring frosts or microbursts [7–9]. These studies generally rely on serendipitous pre-event data, lack true controls and rarely have immediate post-storm impact assessments. Further, the historical understanding of ecosystem response to extreme events based on these *post facto* studies may not be useful in predicting responses to future events, as the underlying template of climate, nutrient reserves, or ecosystem health may have changed over the intervening years [10].

As an alternative to *post facto* analyses, a growing number of controlled experiments are emerging to help elucidate and model ecosystem responses to changes in the frequency, intensity and severity of extreme events [e.g., 11, 12]. These experiments allow for investigation of cause and-effect relationships with rigorous statistical models. They typically include pre- and post-extreme event conditions as well as multiple levels and frequencies of disturbance that allow for determination of response thresholds (i.e., the level of disturbance when an ecosystem irreversibly shifts from one state to another) [4, 6, 13]. Experiments also have the advantage that they can be implemented under controlled conditions designed to improve safety associated with working in hazardous conditions.

Ice storms are a type of extreme winter weather event that are a major driver of forest disturbance in temperate and boreal ecosystems worldwide [14–18]. Climate modeling studies suggest that these types of winter storms will become more frequent and/or more intense under a changing climate [19, 20]. Impacts of ice storms on forests depend on a variety of factors including amount of ice accretion, stand age, stand health and species composition [21]. Direct impacts range from twig and branch breakage with light-to-moderate icing, to loss of tree tops and uprooting of entire trees with severe icing [16, 22–24]. Based on *post facto* studies, these changes can lead to a cascade of changes in forest ecosystem structure and function, including increased inputs of coarse and fine woody debris to the forest floor, increases in light below the forest canopy, reductions in photosynthetic capacity and stored plant carbohydrates, alterations of soil temperature and moisture, changes in belowground plant and microbial processes, and altered leaching and gaseous nutrient losses from the soil [5, 8, 25–27].

Despite this general understanding of the impacts of ice storms on forest ecosystems, manipulation of icing intensity and frequency followed by investigation of ecosystem effects have not been attempted to date. Given the widespread occurrence of these extreme weather events in north temperate and boreal regions, their immediate and lasting impacts, and projections for increased frequency and intensity under a changing climate, there is an urgent need to better understand the impacts of a range of ice loadings and return intervals on forest ecosystems.

The objectives of this paper are to (1) provide a detailed methodological approach for creating experimental ice storms, (2) discuss the efficacy of the design (i.e., our ability to create glaze ice on surfaces), (3) describe possible artifacts of the treatments, and (4) present results on the initial impact of these experimental storms on nutrient transfers in fine and coarse woody debris and tree canopy damage assessments. This work was conducted at the Hubbard Brook Experimental Forest (HBEF) in New Hampshire, builds on a feasibility study at the same site [17], and complements reporting by Fahey et al. [28] on the response of forest canopy structure to these experimental perturbations. Fahey et al. [28] detailed how experimental ice storms increased canopy openness, light transmission and complexity. Here, we describe the initial loss of wood and nutrients from the canopy that contributed to those changes in canopy structure. The detailed description of the methods and initial results on fine and coarse woody debris inputs and tree canopy damage will aid in the design of future studies on ice storm impacts specifically, and extreme weather events more generally, and inform utility companies, emergency management organizations, forest managers, municipalities and the public on how to better prepare for future ice storms.

## Materials and methods

### Site description

**Fig 1:**
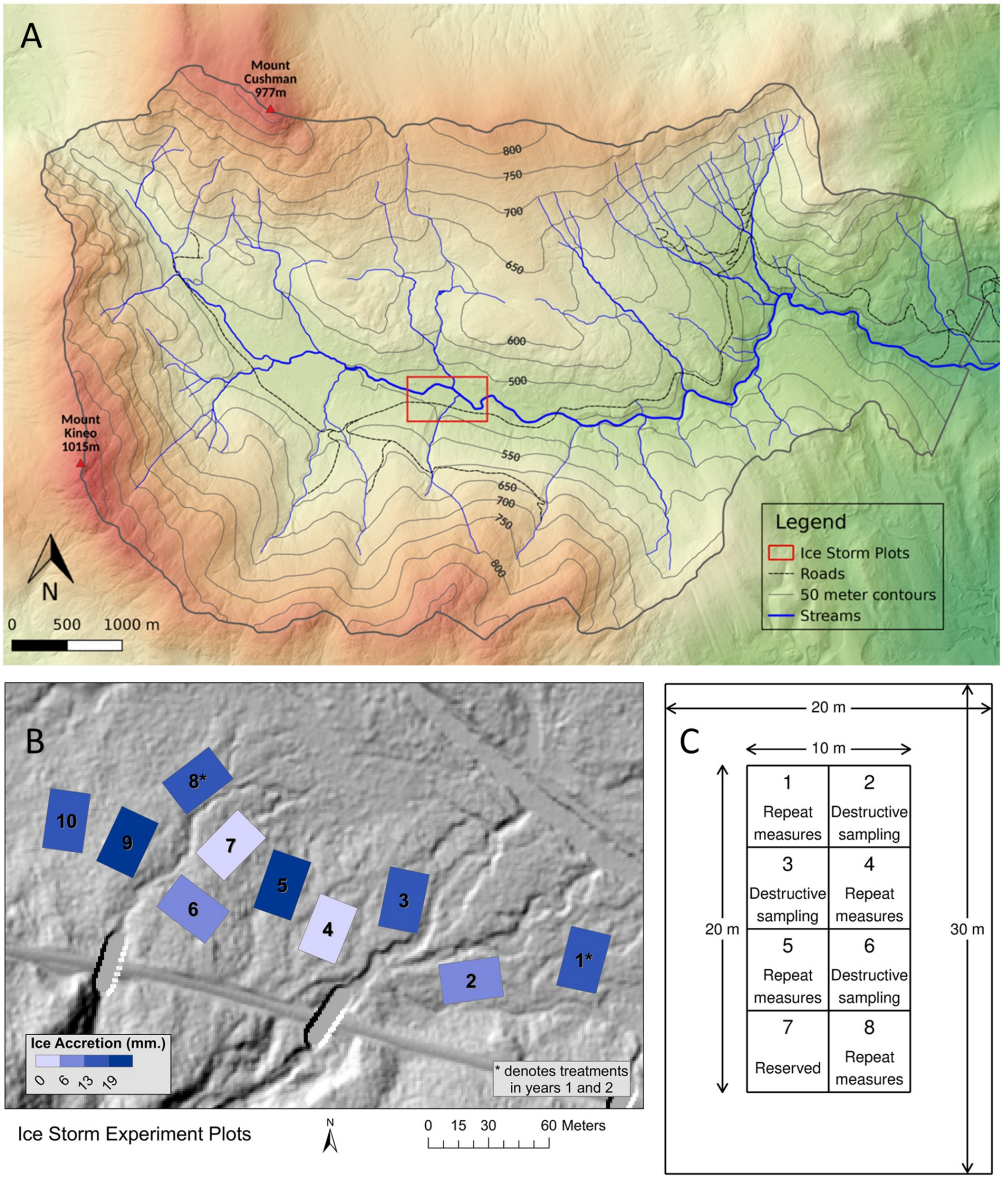
Ice plots location and layout. (A) Experiment location within the Hubbard Brook Experimental Forest. (B) Ice plot location between the Hubbard Brook (top right) and the Hubbard Brook Experimental Forest access road (bottom). Plots 1–5 are in Block 1 and plots 6–10 are in Block 2. Asterisk indicates plots that were iced in both 2016 and 2017. (C) Individual ice plot design, showing the entire plot (20 x 30 m), the inner plot (10 x 20 m), and subplots (5x5 m) for destructive and non-destructive measurements.

The HBEF is a 3,160-ha valley in central New Hampshire, USA (43°56’46”N, 71°47’19”W) (Fig 1A). The climate is humid continental with mean monthly air temperatures ranging from -9 °C in January to 18°C in July [29]. Annual precipitation averages 1400 mm (30% as snow) and is distributed relatively evenly throughout the year. Much of the Hubbard Brook Valley was cut approximately 100 years ago, with subsequent impacts from a hurricane in 1938 and an ice storm in 1998.

**Fig 2:**
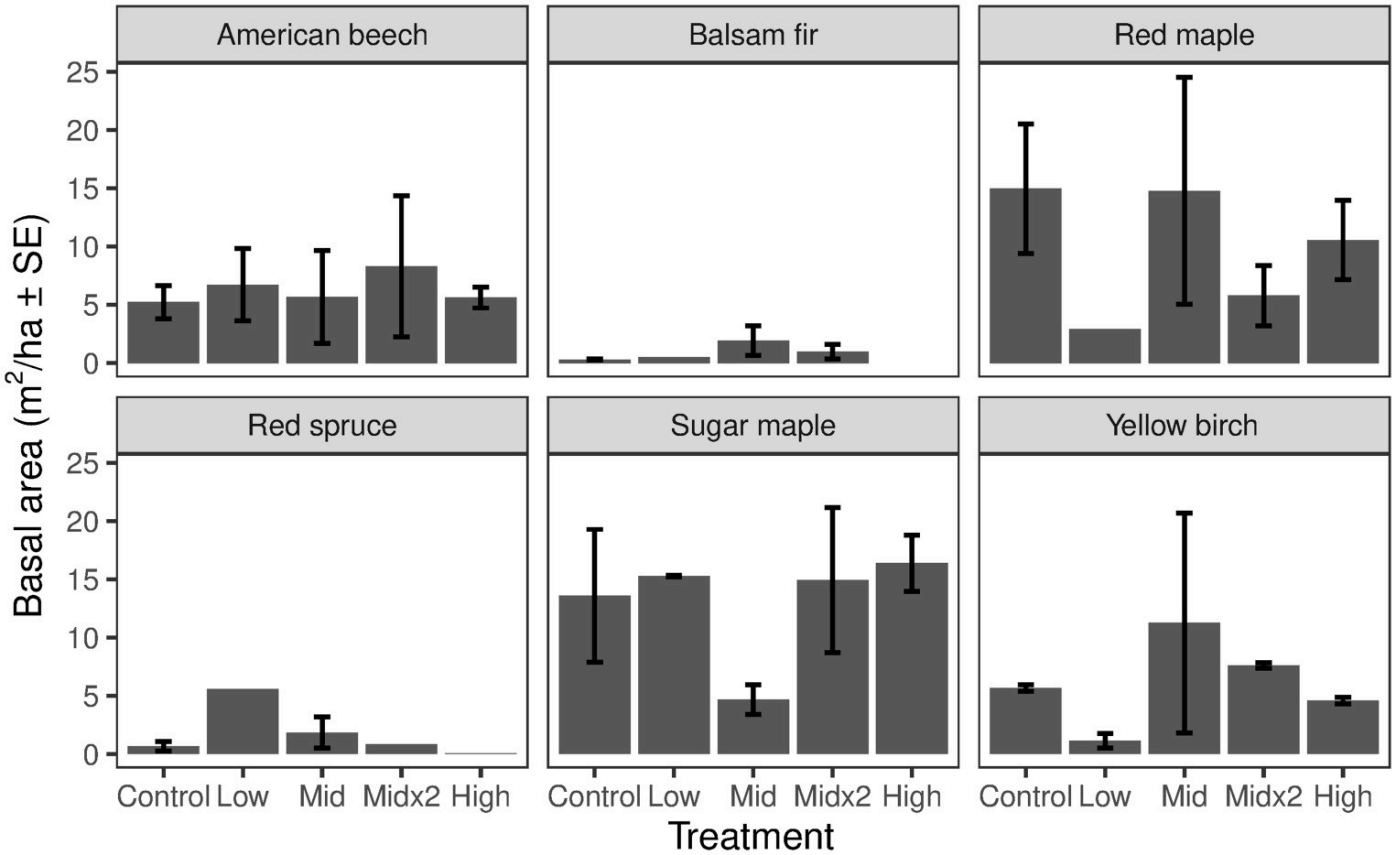
Pre-treatment tree basal area for all trees ≥ 5 cm DBH by species and treatment. Treatments did not differ in mean basal area for any species (*P* > 0.05). Vertical lines are ± 1 standard error.

The ice storm experiment was conducted in the central portion of the Hubbard Brook Valley (~520 m altitude; 43°56’09”N, 71°45’51”W; Fig 1a). Total basal for all trees ≥ 5cm D.B.H. within the experimental plots was 37.9 m^2^/ha and was comprised of eight species: sugar maple (*Acer saccharum* Marsh.) (34%), red maple (*Acer rubrum* L.) (25%), American beech (*Fagus grandifolia* Ehrh.) (17%), yellow birch (*Betula alleghaniensis* Britton) (16%), red spruce (*Picea rubens* Sarg.) (3%), bigtooth aspen (*Populus grandidentata (Michx*.*)* (2%), balsam fir (*Abies balsamea* (L.) Mill.) (2%) and paper birch (*Betula paprifera* Marsh.) (1%) (Fig 2). Canopy height was ~20 m and stand age was estimated at 70–100 years in 2015, with the stand likely originating from the 1938 hurricane. No significant differences in basal area were observed across the treatment plots for any species at the start of the study (Fig 2).

### Experimental design

Ten 20×30 m plots were established in May 2015 and were blocked by location, with plots 1 through 5 in one block on the eastern side of the study site, and plots 6 through 10 on the western side (Fig 1B). Within the block, plots were randomly assigned to one of five icing treatments, resulting in two replicate plots per treatment (Fig 1B). Treatments were based on target radial ice accretion, which is defined as the radius of ice added to a cylindrical structure such as a wire or branch [30]. Treatments (in mm radial ice accretion) included Control (0 mm), Low (6.4 mm), Mid (12.7 mm), High (19.1 mm), and Mid×2 (12.7 mm applied in two consecutive winters). The Low and Mid treatments were chosen to correspond to National Weather Service Ice Storm Warnings, which are 6.4 mm for mid-Atlantic states and 12.7 mm for New York and New England [31]. The High treatment was chosen to represent an extreme ice storm comparable to the 1998 storm [8].. The minimum distance between plots was 10 m. The rectangular plot shape maximized the ability to ice the interior of plots from outside the plots.

The entire 20×30-m plot was iced during treatments. All response measurements were made within an inner 10×20-m area, allowing for a 5-m buffer zone (Fig 1C). The inner 10×20-m plot was further subdivided into eight 5×5-m subplots (Fig 1C). Four of these subplots were used for non-destructive measurements repeated over time; three were used for destructive measurements; and one was undisturbed and reserved for future measurements.

### Ice application

To create ice, stream water (~4°C) was taken from Hubbard Brook by two low pressure/high volume pumps and supplied to two track-equipped utility terrain vehicles (UTV) via moveable fire hoses. Each UTV was equipped with a BB4^®^ centrifugal pump that fed high pressure water to a nozzle mounted next to the pump. Pump nozzles were pressurized to flow at approximately 300 liters per minute. All pumps were gasoline powered. One UTV operated along each of the two long sides of the plot, with each spraying water the full length and half the depth of the plot (Fig 1C; S1 Fig). Water was sprayed up through gaps in the tree canopy, with spray nozzles adjusted so that the water would be deposited over the canopy as a fine mist, freezing on contact with vegetation surfaces. The two UTV teams worked simultaneously, spraying layers of ice on the canopy from opposite sides of the plot. The total amount of time of water application was recorded for each UTV ensemble, with approximately equal icing times on both sides. Icing continued until visual inspection of branches within the plots and physical measurements of ice accretion on twigs indicated that the target ice accretion had been achieved. Final measurements of ice accretion were made immediately following the icing treatments on passive ice collectors, as described below.

#### Weather conditions during icing

Air temperature was measured using two Campbell Scientific^®^ HMP 60 thermistors housed in gill solar radiation shields, with one located near plot 9 and one near plot 2. Wind speed was measured with a RM Young^®^ model 05103 anemometer at a weather station approximately 5 km east of the experimental plots (250 m altitude). Application dates were chosen to meet criteria assuring effective ice accumulation on canopy surfaces (air temperature < -4°C) with minimal drift of mist (i.e. wind speed < 4.5 m/s) as detailed by Rustad and Campbell [17]. Much of the applications took place at night when low temperature requirements were met.

#### Water volume and chemistry of application

Volume of water applied per plot was calculated as the product of application time and water flow. To construct chemical budgets for the treatments, a 60 ml aliquot of Hubbard Brook stream water was collected prior to each icing, and analyzed for sulfate (SO_4_), nitrate (NO_3_) and chloride (Cl) on a Metrohm^®^ Ion Chromatograph; calcium (Ca), magnesium (Mg), potassium (K), and sodium (Na) on an Agilent^®^ 730 ICP optical emission spectrometer; dissolved organic carbon (DOC) and total dissolved nitrogen (TDN) on a Shimadzu^®^ TOCV with a TNM-1 nitrogen (N) detector; and ammonium (NH_4_) by colorimetry on a SEAL Analytical AQ2 discrete analyzer at the Louis C. Wyman Forest Sciences Laboratory, Durham, NH. Dissolved organic N (DON) was calculated as the difference between TDN and inorganic N (NO_3_-N + NH_4_-N). Total mass of elements applied to the canopy as iced ‘precipitation’ was calculated as the ‘precipitation’ chemistry from the stream aliquot multiplied by the ‘precipitation’ volume for each plot.

#### Ice accretion

**Fig 3:**
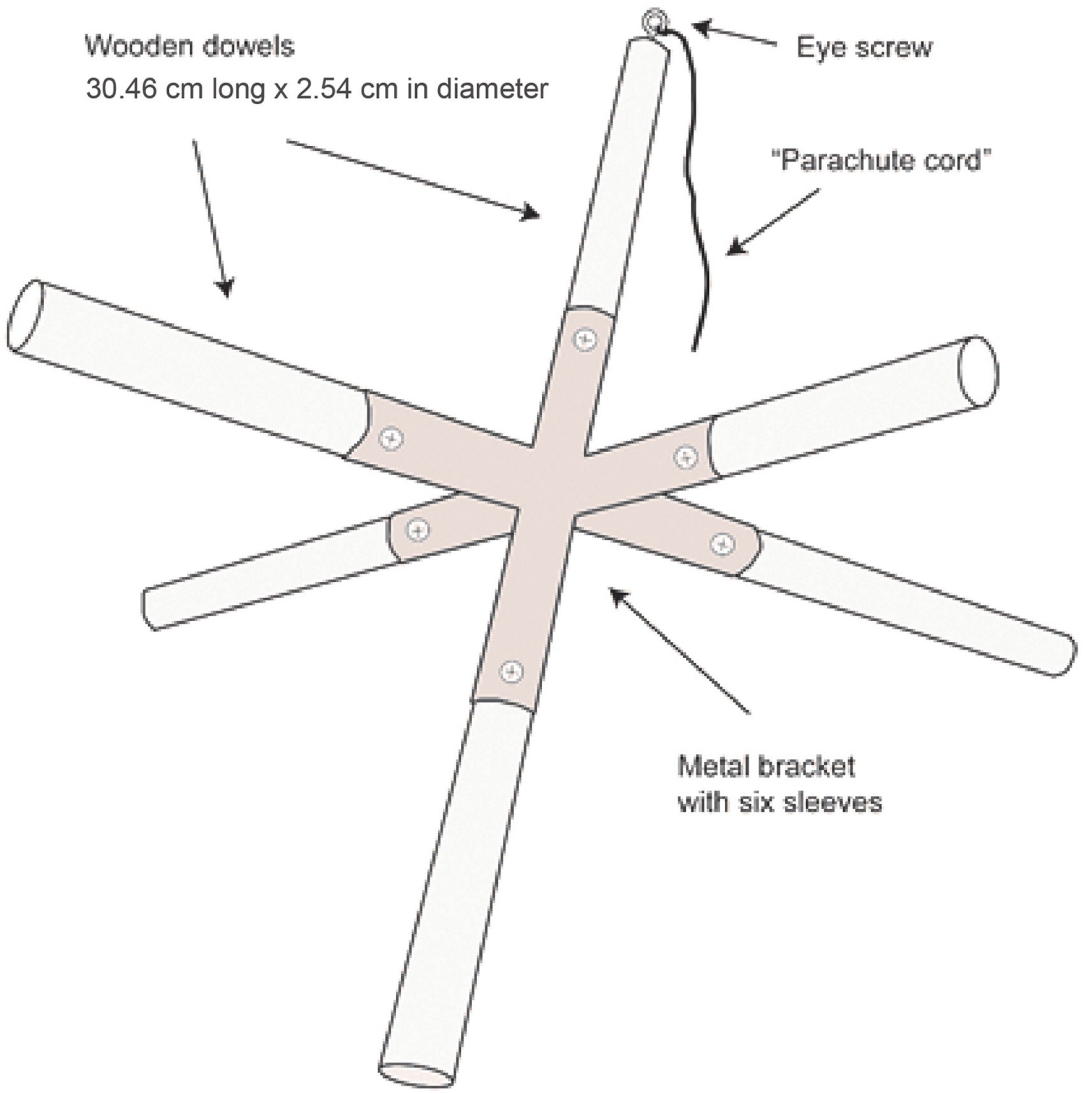
Diagram of the passive ice collector used for measuring ice thickness.

Ice accretion was measured using passive collectors constructed from 2.54 cm diameter birch dowels, with six 30-cm long arms radiating out from a central six-way galvanized steel connector in six directions (0°, 90°, 180°, 270°, up and down; Fig 3). Four collectors were suspended in the forest canopy using parachute cord (to provide a low resistance surface to facilitate release after icing) at a height of ~15 m prior to the icing, with one collector in each of the four cardinal corners of the inner plots.

Radial ice accretion on collectors was measured with two methods. First, ice thickness was measured in the field immediately following icing with digital calipers at multiple points along the six dowel arms. Second, after ice thickness measurements were made in the field, each of the six arms of the collectors were cut at the base with a reciprocating saw, being careful to keep the ice on the dowel intact. Ice-coated dowels were placed individually in labeled zip lock bags and transported back to HBEF Headquarters where the ice was melted at room temperature and the volume of meltwater from each dowel measured. The radial ice accretion equivalent (Req) was calculated using the equation from [30]:

$$Req=-\frac{D}{2}+\sqrt{\left(\frac{D^{2}}{4}+\frac{V}{\pi \rho_{i}L}\right)}$$

where *D* = diameter of the dowel (cm), *L* = length of the dowel (cm); *V* = volume of melted ice (mL), and ρ_i_ = density of glaze ice (g cm^-3^).

### Canopy throughfall volume and chemistry

Canopy throughfall volume was measured with 20 L (28.7 cm diameter) buckets placed on the ground in the center of each subplot (eight per plot). Two of these buckets were acid washed and designated for chemical analyses. After icing, buckets were retrieved, thawed, and the volume of canopy throughfall in each bucket was measured. Aliquots from the two acid-washed buckets per plot were poured off, refrigerated, and analyzed for SO_4_, NO_3_, Cl, Ca, Mg, K, Na, NH_4_, DOC, TDN and DON by the same methods as for the stream water aliquots. Total throughfall flux for each element in each plot was calculated as the mean plot chemistry multiplied by the mean plot volume.

### Fine and coarse woody debris

Fine woody debris (i.e., woody material < 2 cm in diameter; hereafter FWD) and foliar litter were collected in plastic baskets (52L×37W×27H cm) that were placed in the center of each of seven subplots for all plots. Foliar litter was collected during the pre-treatment period 5 August, 2015 to 17 January, 2016. Fine woody debris was collected between two to four weeks after icing treatments in 2016 and ten weeks after icing treatments in 2017 to allow all the near-term broken branches to reach the ground. In instances where larger branches lay on the litter baskets, twigs less than 2 cm were clipped around the perimeter of the basket and were included as part of the sample. All foliar litter and FWD samples were oven dried at 60°C to constant weight, weighed, ground through a Wiley Mill^®^ #40 mesh sieve, and analyzed for total carbon (C) and nitrogen (N) on a Thermo Flash^®^ EA 1112 Series CN Analyzer^®^ and for total Ca, Mg, K, and phosphorus (P) by microwave digest and analysis on an ICP [32]at the Louis C. Wyman Forest Sciences Laboratory, Durham, NH. Total mass of all elements was calculated as total litter mass multiplied by element concentration.

Coarse woody debris (i.e., woody material ≥2 cm diameter; hereafter CWD) was collected on a 2m×2m quadrat located within each of the three subplots designated for destructive measurements on each of the main plots (Fig 1C). On November 16, 2015, prior to icing, all CWD was collected from these quadrats. Post-treatment CWD was collected in July and November of 2016 and 2017. Downed branches and tree boles that crossed quadrat boundaries were cut on the border with a hand saw. Coarse woody debris was sorted into five decay classes as described in Table 1 [after 33]. The wet weight of CWD for all categories was measured in the field for each quadrat. A subsample comprised of approximately twenty percent of the total wet weight of CWD for each category was brought back to the laboratory and oven-dried at 60°C to a constant weight to obtain oven dry mass and a moisture correction factor. Samples were ground and analyzed for nutrients by the same method as for FWD. Total mass of all elements was calculated as total litter mass multiplied by element concentration.

**Table 1:** Decay class designations for coarse woody debris [after 33].

Class	Structural Integrity	Texture	Color	Invading Roots	Twigs + Branches
1	Sound	Intact, no rot, conks on stem absent	Original color	Absent	If branches present, fine twigs still attached with tight bark
2	Sapwood somewhat decayed	Mostly intact, sapwood partially soft, cannot pull apart by hand	Original color	Absent	If branches present, many fine twigs gone, fine twigs present have peeling bark
3	Sapwood decayed, log supports its own weight	Large, hard pieces of sapwood can be pulled apart by hand	Red-brown or original color	Present in sapwood only	Large branch stubs will not pull out
4	Heartwood rotten, does not support weight, shape maintained	Soft, small, blocky pieces, metal pin can push apart heartwood	Red-brown or light brown	Present throughout log	Large branch stubs pull out easily
5	No structural integrity, does not maintain shape	Soft, powdery when dry	Red-brown to dark brown	Present throughout log	Branch stubs and pitch pockets have rotted away

### Tree canopy damage assessment

A qualitative tree canopy damage assessment was made pre- and post-treatment, following methods used after the 1998 ice storm and the HBEF pilot ice storm experiment [34]. In this method, all trees greater than 5.0 cm DBH were identified, tagged, measured (DBH, height), and mapped. Basal area was calculated from tree diameter by species. All tagged trees were assigned to an injury class value based on a visual inspection of the branches, using the following crown injury classes: 1 = no visible damage; 2 = 1–49%; 3 = 50–75%; 4 = >75%; 5 = dead. Damage assessments were made on May 12, 2015, August 9, 2016, August 16, 2017, August 7, 2018 and July 1, 2019.

### Statistical analysis

#### Ice, throughfall and precipitation

A series of linear mixed effects models were used to analyze throughfall ice accretion, water volumes and chemistry. Response variables included ice accretion (mm), water volume (mm depth m^-2^) and mass (mg m^-2^) of 10 analytes (Ca^2+^, Mg^2+^, K^+^, Na^+^, Cl^-^, SO_4_^2-^-S, NO_3_^-^-N, NH_4_^+^-N, DON, DOC); explanatory variables included treatment and a random effect for plot. The lme4 package in R was used to conduct mixed effects analyses [35, 36]. As only one sample was collected per plot for precipitation, plot was not used as a random effect for these data. The emmeans package in R was used to conduct multiple comparisons testing and an α of 0.05 was used to assess significance for both throughfall and precipitation [37]. We calculated the amount of ice accretion retained in the canopy as the volume of precipitation minus the volume of throughfall.

#### Fine and coarse woody debris

Linear mixed effect models were used to analyze FWD and CWD total mass, C, N, P, Ca, Mg, and K mass. Response variables included FWD and CWD total mass and nutrients; explanatory variables included treatment (for FWD and CWD), sampling period (for CWD) and treatment × sampling period (for CWD). Plot was incorporated as a random effect. An α of 0.05 was used to compare treatments (for FWD and CWD) and sampling periods (for CWD). Cumulative link mixed models were used to assess decay class as an ordered, categorical variable [38]. CWD mass was used as weights in the decay class models. Differences among treatments were calculated in each sampling period, using treatment as a fixed effect and plot as a random effect. Decay classes from the July 2016 and 2017 samples were compared for Mid×2 plots. Weighted means of decay classes are presented as numbers to illustrate differences among treatments.

#### Tree canopy damage assessment

Cumulative link mixed models were used to assess damage as an ordered, categorical variable. Fixed effects were treatment × sampling period × species; and tree was used as a random effect. Only trees within the inner 10×20 m buffer were included in the analysis. Eight trees were excluded from analyses from one year of sampling (*n*_2017_ = 2, n_2018_ = 6) because they were not found or were not assigned a damage class. Multiple comparisons testing with an α of 0.05 were used to compare treatments and sampling periods. We conducted three rounds of analyses: 1) all species including all treatments, species was not included as a fixed effect in this model; 2) American beech, sugar maple, and yellow birch including all treatments; and 3) American beech, red maple, sugar maple, and yellow birch, including the Control, Mid, Mid×2, and High treatments as there were no red maple trees in the Low treatment. Weighted means of damage classes are presented as numbers to illustrate differences among treatments, sampling periods and species.

## Results

### Weather conditions during icing

Air temperature and wind speed during the spray applications were generally within the target range of < -4°C and < 4.5 m/s (Table 2). Mean temperatures across the plots during spray applications ranged from -12.1 to -5.3°C, and mean wind speeds across the plots ranged from 0.23 to 1.2 m/s.

**Table 2:** Weather conditions during spraying for 2016 and 2017 ice applications, and estimated amounts of water applied to plots retained in the canopy as ice.

*Plot*	Start Date	Start Time	End Date	End Time	Mean Temp (°C)	Max Temp (°C)	Min Temp (°C)	Mean Wind Speed (m/s)	Max Wind Speed (m/s)	Min Wind Speed (m/s)	Percent of Water Retained in Canopy as Ice
*1*	1/18/2016	14:46	1/18/2016	17:20	-10.41	-10.01	-10.83	1.20	1.73	0.87	65%
*2*	2/11/2016	11:10	2/11/2016	12:15	-8.37	-7.51	-9.74	0.96	1.23	0.82	68%
*3*	1/27/2016	21:06	1/28/2016	1:16	-10.36	-7.45	-12.32	0.79	1.00	0.38	45%
*5*	1/28/2016	1:45	1/28/2017	5:28	-14.14	-12.65	-15.53	0.69	0.95	0.39	47%
*6*	2/11/2016	9:00	2/11/2016	10:03	-8.03	-7.84	-8.22	0.93	1.27	0.59	48%
*8*	1/18/2016	18:25	1/18/2016	20:25	-12.10	-11.36	-12.90	1.34	1.77	1.03	47%
*9*	1/28/2016	20:22	1/29/2016	0:55	-5.29	-3.72	-5.95	0.57	0.80	0.13	55%
*10*	1/29/2016	1:29	1/29/2016	3:49	-6.87	-6.46	-7.30	0.67	0.82	0.27	50%
*1*	1/14/2017	16:03	1/14/2017	19:03	-10.34	-9.12	-11.02	0.23	0.60	0.02	9%
*8*	1/14/2017	20:33	1/14/2017	23:20	-9.20	-6.79	-12.09	0.47	0.69	0.12	42%

Air temperatures are from sensors at the plots; wind speeds are from Hubbard Brook Experimental Forest weather station 1, approximately 5 km from the treatment plots.

### Water and chemical inputs

Because stream water used for the applications had greater concentrations of most chemical constituents than ambient precipitation at Hubbard Brook, inputs of many chemicals (especially base cations, Cl and SO_4_) exceeded those of natural ice storms and comprised inputs comparable in magnitude to annual precipitation inputs at the site (Table 3). Chemical inputs generally increased with increased treatment applications. Throughfall water volume and chemical constituent inputs were generally lower than those for precipitation water and chemical constituent inputs (reflecting water and chemical retention in the canopy as ice) and followed similar patterns to those described for precipitation volume and chemical constituents (Table 3).

**Table 3:** Volume (mm) and chemistry (mg m^-2^) of precipitation and throughfall for different levels of treatment in the ice storm experiment as compared to mean annual precipitation (2012–2016) and the ice storm of 1998 in the reference watershed (W6) at the Hubbard Brook Experimental Forest.

	Water	Ca^2+^	Mg^2+^	K^+^	Na^+^	Cl^-^	SO_4_^2-^-S	NO_3_^-^-N	NH_4_^+^-N	DON	DOC
Measurement	mm	mg m^-2^									
*Precipitation*											
annual average	1350	100	23	73	192	260	203	196	131	82	1409
1998 ice storm	85	2	<1	1	2	4	16	13	3	<1	17
ISE (Low)	63 d	90 c	20 c	11 c	54 c	28 c	56 c	6 c	<1 c	4 b	133 c
ISE (Mid)	125 bc	174 b	43 b	27 b	127 b	51 b	114 b	11 b	<1 b	5 b	257 b
ISE (Midx2-yr 1)	110 c	124 bc	31 bc	20 b	107 b	49 b	95 b	8 bc	<1 b	4 b	251 b
ISE (Midx2-yr 2)	158 b	141 bc	45 b	22 b	106 b	78 a	133 b	9 bc	1 a	10 a	469 a
ISE (High)	216 a	300 a	73 a	46 a	219 a	89 a	197 a	19 a	1 a	9 a	444 a
*Throughfall*											
ISE (Low)	30 b	46 c	10 c	12 c	34 d	21 b	31 d	4 ab	<1 ab	3 c	90 c
ISE (Mid)	66 b	116 bc	26 bc	29 ab	82 bc	38 b	75 bc	4 ab	<1 ab	5 bc	232 bc
ISE (Midx2-yr 1)	37 b	54 c	12 c	15 bc	39 cd	18 b	36 cd	3 b	<1 b	2 c	102 c
ISE (Midx2-yr 2)	119 a	128 ab	40 ab	32 a	88 b	68 a	110 ab	9 a	1 a	9 a	414 a
ISE (High)	116 a	193 a	46 a	42 a	147 a	67 a	129 a	6 ab	1 ab	8 ab	388 ab

Different letters indicate significant differences among the treatments (*P* < 0.05).

### Canopy water retention and ice accretion

During the first winter of treatments (2016), 45 (Plot 3) to 68 (Plot 2) percent of the water sprayed onto plots was retained by the canopy as ice (Table 1). During the second winter of treatments (2017), 9 (Plot 1) and 42 (Plot 8) percent of the water sprayed onto plots was retained by the canopy as ice (Table 1).

**Fig 4:**
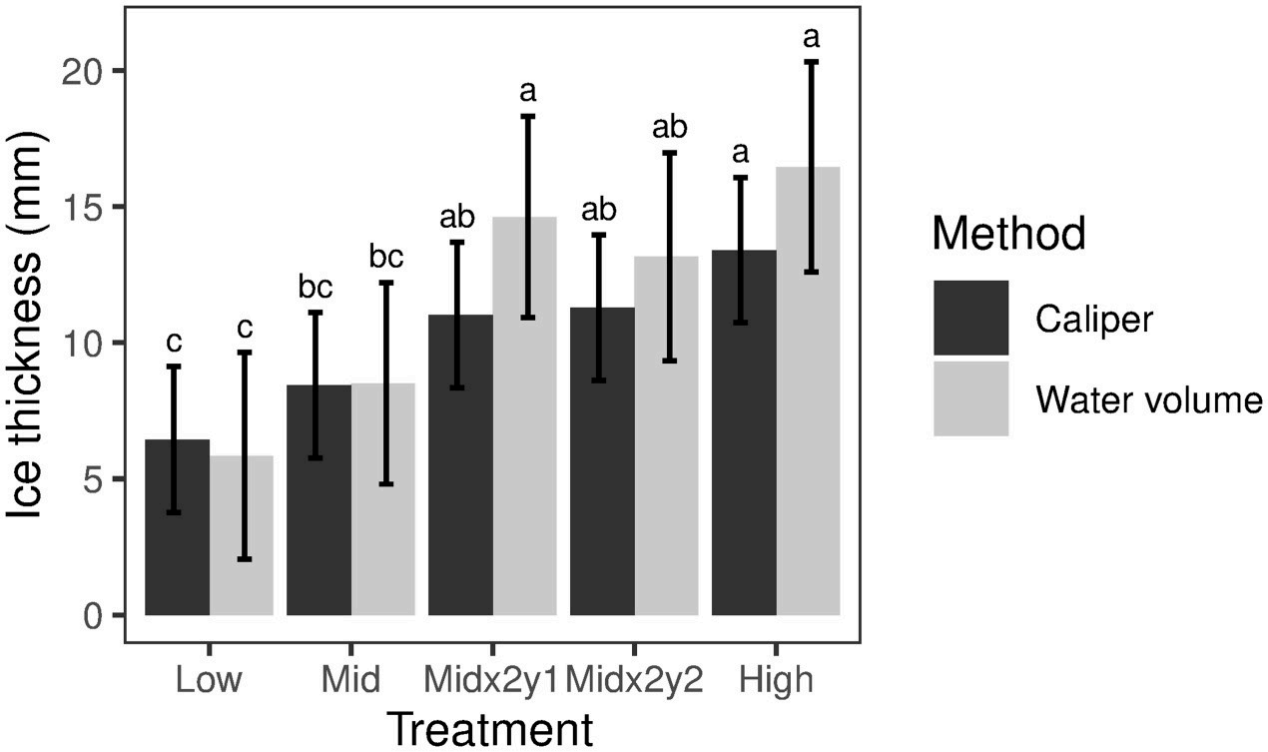
Measured ice thickness using calipers and water volume methods. Different letters denote significant differences among treatments (*P*<0.05). Vertical lines are ±1 standard error.

The two methods for measuring ice accretion were in close agreement for smaller treatments (< 10 mm) with few icicles. Specifically, ice accretion measured by calipers was 111 and 99 percent of ice accretion measured by melted water volume for the Low and Mid treatments, respectively (Fig 4). However, when estimated ice accretion was >10 mm, and icicles were more conspicuous, ice accretion measured by calipers was 75, 85 and 82 percent of ice accretion measured by melted water volume for Mid×2-yr1, Mid×2-yr2 and High treatments, respectively (Fig 4).

In general, the Low, Mid×2-yr1 and Mid×2-yr2 treatments were close to target levels (89 to 99 percent, 87 to 115 percent, and 89 to 104 percent of target values based on caliper and volume estimates, respectively), whereas the Mid and the High treatments were below targets (66 to 67 percent and 70 to 86 percent of target values for caliper and volume estimates, respectively; Fig 4). Across all treatments, measured ice accretion showed a stepwise increase in values from Low- to Mid- to High ice accretion targets, resulting in a suite of experimental light to moderate to severe ice storms (Fig 4).

### Fine woody debris

Mean pre-treatment inputs of foliar litter across the treatments ranged from 1.67 to 310 g m^-2^ for total mass, 0.79 to 147 g m^-2^ for C, 0.01 to 4.61 g m^-2^ for N, 1.12 to 454 mg m^-2^ for P, 0.01 to 3.75 g m^-2^ for Ca, 1.49 to 659 mg m^-2^ for Mg and 5.20 to 1201 mg m^-2^ for K. There were no significant pre-treatment differences in foliar litter mass and nutrients among treatments.

**Fig 5:**
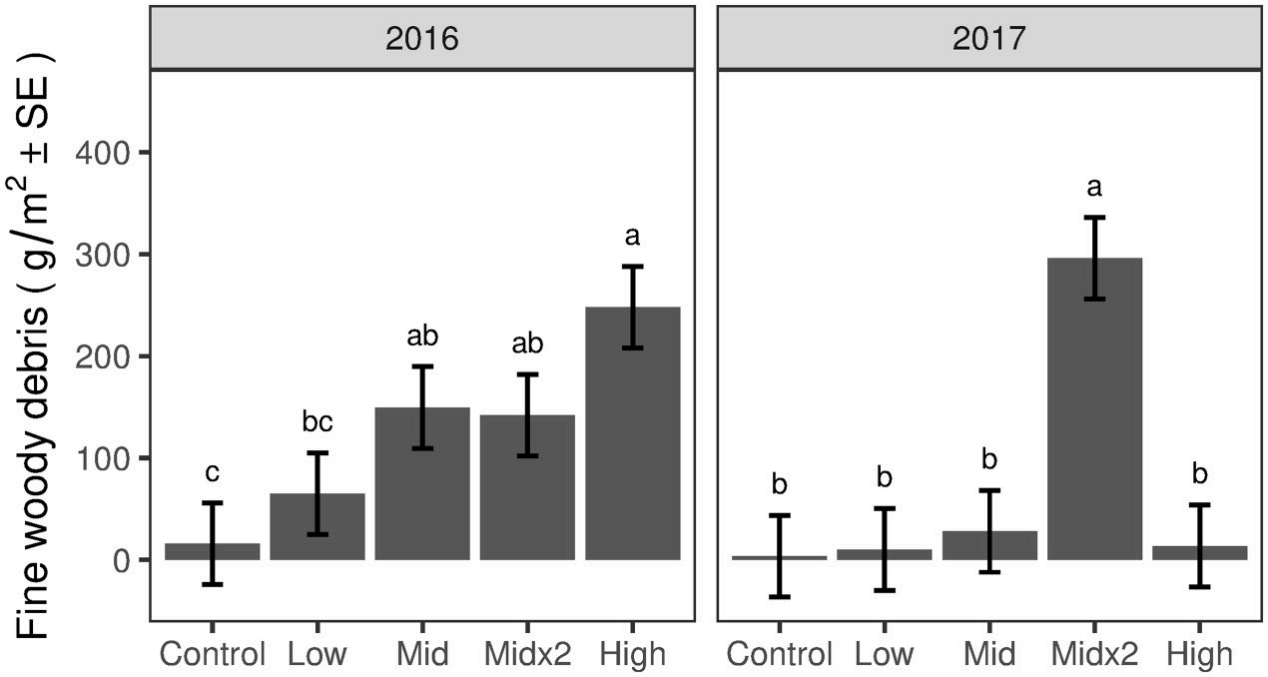
Post-icing FWD mass for winters 2016 and 2017. Different letters denote significant differences among treatments (*P*<0.05) within a sampling period. Vertical lines are 95% confidence intervals.

In February 2016, the first ice applications resulted in a general stepwise increase in the FWD from the Control to the High treatment plots (Fig 5). Statistically, the amounts of FWD deposited on the Mid and Mid×2 treatment plots were greater than the amount deposited on the Control treatment plots; and the amount of FWD deposited on the High treatment plots was greater than the amount deposited on the Low and Control treatment plots (Fig 5). In 2017, following the second ice applications on the Mid×2 treatment plots, significantly greater FWD was deposited on the Mid×2 treatment plots compared to all other treatments (Fig 5). Notably, the FWD deposited on the Mid×2 treatment plots was 2.1 times greater after the 2017 treatment compared to the 2016 treatment (*P* < 0.01).

The mass of FWD nutrients generally followed the same patterns as for total mass for both years. (Table 4).

**Table 4:** Mass of fine woody debris nutrient elements during each sampling period in the winters of 2016 and 2017.

Nutrient	Year	Control	Low	Mid	Midx2	High
C (g m^-2^)	2016	7.88 ± 3.2 (c)	30.54 ± 11.39 (bc)	69.86 ± 18.29 (ab)	66.98 ± 10.53 (b)	120.23 ± 33.81 (a)
C (g m^-2^)	2017	1.77 ± 0.45 (b)	5.09 ± 2.39 (b)	14 ± 5.45 (b)	144.92 ± 44.83 (a)	6.8 ± 2.64 (b)
N (g m^-2^)	2016	0.13 ± 0.06 (c)	0.26 ± 0.06 (bc)	0.99 ± 0.22 (b)	1.01 ± 0.17 (b)	1.92 ± 0.53 (a)
N (g m^-2^)	2017	0.03 ± 0.01 (b)	0.06 ± 0.02 (b)	0.19 ± 0.06 (b)	2.24 ± 0.82 (a)	0.12 ± 0.03 (b)
P (mg m^-2^)	2016	8.43 ± 3.57 (b)	14.51 ± 3.26 (b)	71.8 ± 19.54 (a)	69.65 ± 15.97 (a)	122.27 ± 33.91 (a)
P (mg m^-2^)	2017	1.71 ± 0.36 (b)	3.43 ± 1.38 (b)	9.19 ± 3.05 (b)	205.25 ± 78.67 (a)	5.74 ± 1.29 (b)
Ca (g m^-2^)	2016	0.09 ± 0.03 (c)	0.48 ± 0.16 (bc)	1.13 ± 0.25 (b)	1.15 ± 0.18 (b)	2.02 ± 0.57 (a)
Ca (g m^-2^)	2017	0.04 ± 0.02 (b)	0.05 ± 0.02 (b)	0.14 ± 0.05 (b)	1.97 ± 0.69 (a)	0.11 ± 0.04 (b)
Mg (mg m^-2^)	2016	8.97 ± 3.58 (c)	28.67 ± 7.45 (bc)	81.25 ± 19.64 (b)	85.17 ± 18.75 (ab)	149.7 ± 43.67 (a)
Mg (mg m^-2^)	2017	2.31 ± 0.57 (b)	4.38 ± 1.46 (b)	14.24 ± 5.99 (b)	179.7 ± 58.73 (a)	8.78 ± 2.3 (b)
K (mg m^-2^)	2016	21.13 ± 8.9 (c)	48.38 ± 13.85 (bc)	259.37 ± 85.65 (a)	212.86 ± 39.36 (ab)	368.95 ± 102.54 (a)
K (mg m^-2^)	2017	4.16 ± 1.15 (b)	12.51 ± 5.81 (b)	25.3 ± 9.78 (b)	698.85 ± 268.91 (a)	17.77 ± 4.25 (b)

Letters signify differences among treatments within a sampling period using *P* < 0.05.

### Coarse woody debris

Total mass and element content of CWD removed from the plots in November 2015 was highly variable, reflecting the localized history of inputs. Across all treatment plots, mean CWD mass ranged from 475.08 to 1564.05 g m^-2^, mean C mass ranged from 225.79 to 750.79 g m^-2^, mean N mass ranged from 1.48 to 2.72 g m^-2^, mean P mass ranged from 0.07 to 0.12 g m^-2^, mean Ca mass ranged from 1.75 to 4.19 g m^-2^, mean Mg mass ranged from 0.12 to 0.36 g m^-2^, and mean K mass ranged from 0.20 to 0.55 g m^-2^. No significant pre-treatment differences were observed among the treatment plots for total mass or any of the elemental masses. Weighted mean decay class across all treatment plots was 3.5, with no significant differences among the treatments.

**Fig 6:**
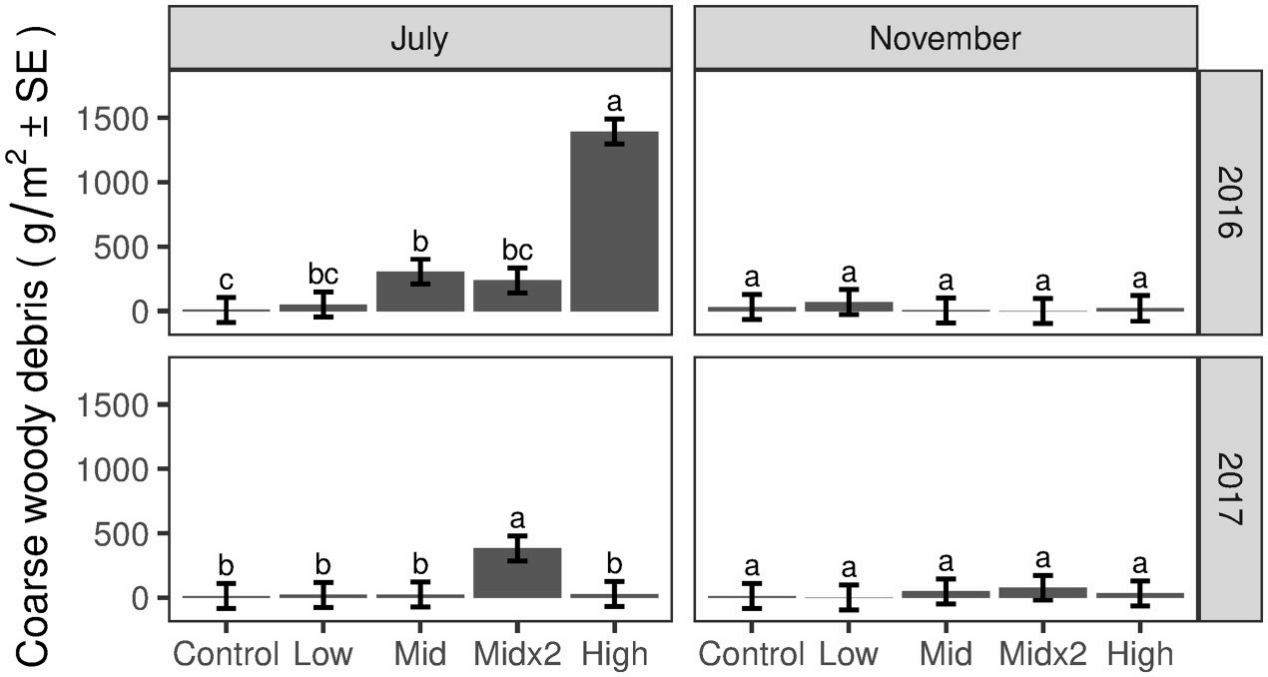
Post-icing coarse woody debris mass for July and November 2016 and 2017. Different letters denote significant differences among treatments (*P*<0.05) within a sampling period. Vertical lines are 95% confidence intervals.

In July 2016, the first ice applications resulted in a general stepwise increase in CWD mass from the Control to the High treatment plots (Fig 6). Significantly greater CWD mass was deposited on the Mid treatment plots compared to the Control plots, and significantly greater CWD mass was deposited on the High treatment plots compared to the other treatment plots (Fig 6). Mean decay classes differed significantly among treatments. Decay classes were lowest (i.e., the wood was least decayed) for the High and Mid×2 treatment plots (weighted mean decay classes = 1.3 for both), mid-range for the Mid treatment plots (weighted mean decay class = 1.7), and highest for the Control and Low treatment plots (weighted mean decay classes = 2.3 and 2.6, respectively). Little additional CWD fell between July and November 2016 (Fig 6), and the sample size (only 12 quadrats total with measurable CWD) was too low for meaningful statistical comparisons.

Following the second year of ice applications to the Mid×2 plots, a larger mass of CWD was deposited on the Mid×2 plots compared to the other treatment plots (July 2017, Fig 6). This amount was similar to the CWD mass deposited after the 2016 Mid and Mid×2 ice applications. Mean decay classes differed significantly among the treatments. Decay classes were lower in the High, Mid×2 and Mid treatment plots (weighted mean decay classes = 2.6, 2.1, and 2.4, respectively) compared to the Control and Low treatment plots (weighted mean decay classes = 3.7 and 3.6, respectively). For the Mid×2 treatments, decay classes were greater for CWD collected in July 2017 compared to CWD collected in July 2016 (weighted mean decay class = 2.08 vs 1.27, respectively; *P* < 0.001). Little additional CWD fell between July and November 2017 (Fig 6). Decay classes differed significantly among the treatments, with mean decay class in Mid and Mid×2 treatment plots (weighted mean decay classes = 2.7 and 2.5, respectively) lower than the Control plots (weighted mean decay class = 3.6). The Mid treatment had a lower decay class than the High treatment plots as well (weighted mean decay classes = 2.7 and 3.6, respectively). Thus, nearly all the CWD resulting from the experimental ice applications fell during and immediately after the applications, the CWD was generally less decayed in the Mid, Mid×2 and High treatment plots compared to the Control and Low treatment plots, and the CWD that fell in 2017 had a greater component of older, more decayed wood than the CWD that fell in 2016.

Total mass of CWD nutrient elements generally followed those of total CWD mass (Table 5).

**Table 5:** Mass of coarse woody debris nutrient elements during each sampling period.

Nutrient	YR_M	Control	Low	Mid	Mid×2	High
C (g m^-2^)	2016_07	4.23 ± 2.13 (c)	23.6 ± 10.69 (bc)	141.58 ± 44.57 (b)	114.49 ± 67.76 (bc)	669.26 ± 181.09 (a)
C (g m^-2^)	2016_11	14.06 ± 7.75 (a)	32.21 ± 23.18 (a)	2.51 ± 1.87 (a)	0 ± 0 (a)	10.02 ± 4.57 (a)
C (g m^-2^)	2017_07	6.28 ± 2.79 (b)	10.61 ± 5.4 (b)	11.46 ± 5.99 (b)	182.62 ± 77.43 (a)	13.57 ± 5.73 (b)
C (g m^-2^)	2017_11	6.15 ± 3.58 (a)	0.94 ± 0.94 (a)	23.4 ± 13.05 (a)	36.48 ± 18.23 (a)	16.23 ± 11.13 (a)
N (g m^-2^)	2016_07	0.03 ± 0.01 (c)	0.17 ± 0.07 (c)	1.28 ± 0.4 (b)	0.98 ± 0.61 (bc)	4.98 ± 1.35 (a)
N (g m^-2^)	2016_11	0.06 ± 0.03 (a)	0.28 ± 0.23 (a)	0.02 ± 0.02 (a)	0 ± 0 (a)	0.06 ± 0.03 (a)
N (g m^-2^)	2017_07	0.04 ± 0.02 (b)	0.1 ± 0.06 (b)	0.1 ± 0.05 (b)	1.83 ± 0.75 (a)	0.11 ± 0.06 (b)
N (g m^-2^)	2017_11	0.04 ± 0.02 (a)	0.01 ± 0.01 (a)	0.15 ± 0.09 (a)	0.24 ± 0.12 (a)	0.11 ± 0.07 (a)
P (mg m^-2^)	2016_07	0.94 ± 0.47 (c)	6.42 ± 3.37 (bc)	73.72 ± 25.86 (b)	53.71 ± 30.91 (bc)	360.31 ± 105.62 (a)
P (mg m^-2^)	2016_11	2.7 ± 1.6 (a)	14.09 ± 9.66 (a)	1.49 ± 1.34 (a)	0 ± 0 (a)	3.5 ± 1.74 (a)
P (mg m^-2^)	2017_07	1.79 ± 0.92 (b)	4.35 ± 2.42 (b)	5.13 ± 3.11 (b)	131.72 ± 56.08 (a)	6.11 ± 3.4 (b)
P (mg m^-2^)	2017_11	2.1 ± 1.22 (a)	0.32 ± 0.32 (a)	7.99 ± 4.46 (a)	12.46 ± 6.23 (a)	5.54 ± 3.8 (a)
Ca (g m^-2^)	2016_07	0.06 ± 0.03 (c)	0.25 ± 0.09 (c)	2.52 ± 0.74 (b)	1.36 ± 0.69 (bc)	7.27 ± 2.33 (a)
Ca (g m^-2^)	2016_11	0.12 ± 0.06 (a)	0.54 ± 0.37 (a)	0.03 ± 0.03 (a)	0 ± 0 (a)	0.09 ± 0.04 (a)
Ca (g m^-2^)	2017_07	0.05 ± 0.02 (b)	0.16 ± 0.08 (b)	0.24 ± 0.16 (b)	3.22 ± 1.41 (a)	0.19 ± 0.08 (b)
Ca (g m^-2^)	2017_11	0.07 ± 0.04 (a)	0.01 ± 0.01 (a)	0.26 ± 0.14 (a)	0.4 ± 0.2 (a)	0.18 ± 0.12 (a)
Ca (g m^-2^)	2018_07	0.11 ± 0.07 (a)	1.26 ± 1.11 (a)	0.11 ± 0.08 (a)	1.02 ± 0.43 (a)	0.63 ± 0.28 (a)
Mg (mg m^-2^)	2016_07	3.43 ± 1.84 (c)	21.53 ± 9.03 (bc)	123.53 ± 39.28 (b)	96.39 ± 53.52 (bc)	564.36 ± 161.88 (a)
Mg (mg m^-2^)	2016_11	10.62 ± 5.8 (a)	51.71 ± 37.77 (a)	1.5 ± 1.33 (a)	0 ± 0 (a)	7.04 ± 3.28 (a)
Mg (mg m^-2^)	2017_07	2.73 ± 0.9 (b)	15.97 ± 11.56 (b)	16.45 ± 12.05 (b)	139.93 ± 58.71 (a)	12.85 ± 5.81 (b)
Mg (mg m^-2^)	2017_11	5.1 ± 2.97 (a)	0.78 ± 0.78 (a)	19.37 ± 10.81 (a)	30.2 ± 15.09 (a)	13.44 ± 9.22 (a)
K (mg m^-2^)	2016_07	5.06 ± 3.62 (b)	21.58 ± 10.11 (b)	284.05 ± 113.26 (b)	318.15 ± 223.18 (b)	1816.3 ± 703.73 (a)
K (mg m^-2^)	2016_11	18.33 ± 9.75 (a)	26.83 ± 17.91 (a)	7.16 ± 6.91 (a)	0 ± 0 (a)	16.61 ± 9.27 (a)
K (mg m^-2^)	2017_07	4.6 ± 2.03 (b)	16.37 ± 9.89 (b)	33.03 ± 24.91 (b)	541.95 ± 230.64 (a)	20.74 ± 10.83 (b)
K (mg m^-2^)	2017_11	9.12 ± 5.31 (a)	1.39 ± 1.39 (a)	34.68 ± 19.35 (a)	54.06 ± 27.02 (a)	24.05 ± 16.5 (a)

Letters signify differences among treatments within a sampling period using *P* < 0.05.

### Tree canopy damage assessments

**Fig 7:**
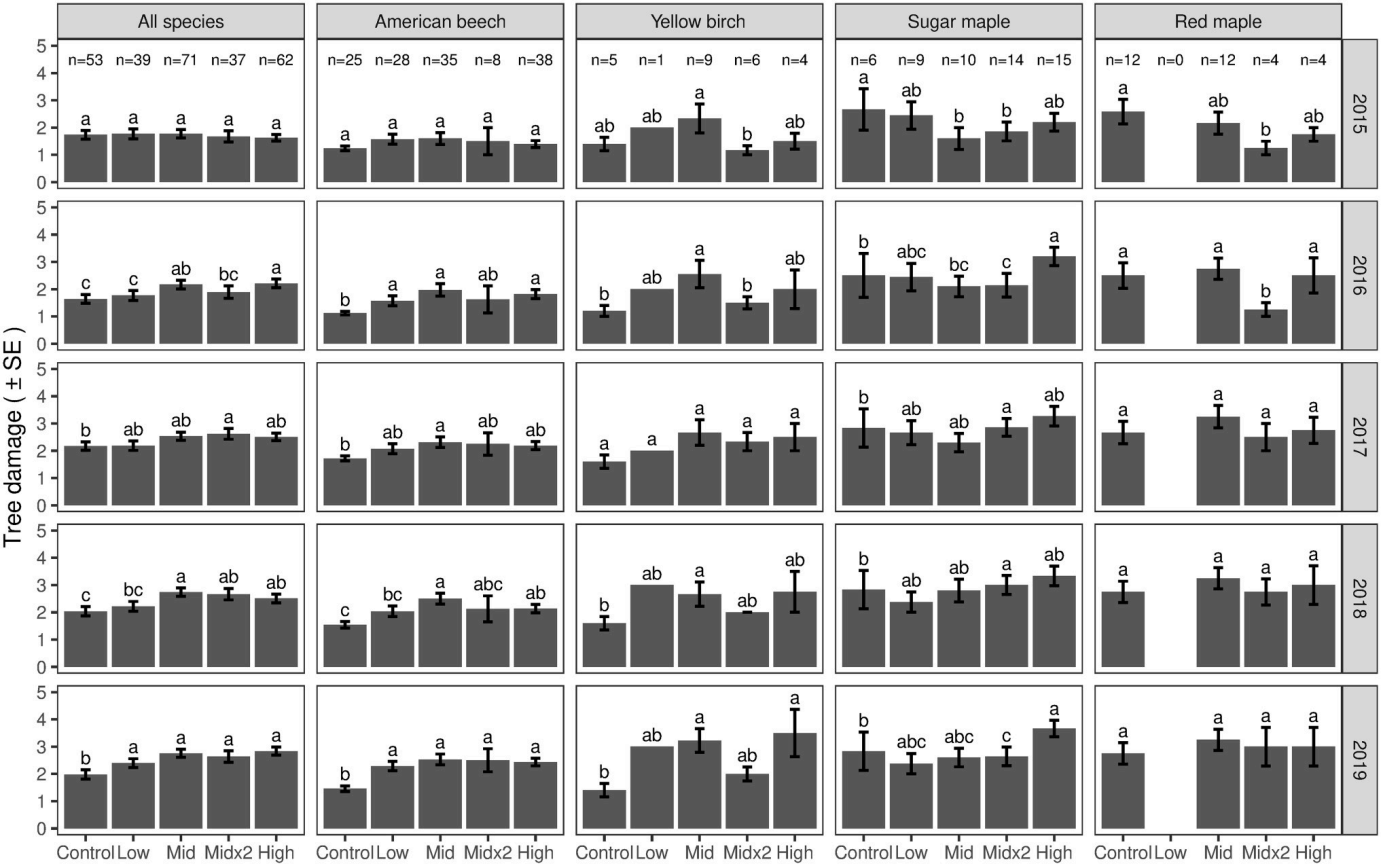
Tree canopy damage assessments among treatments grouped by year. Different letters denote significant differences among treatments (*P*<0.05) within a sampling period. Vertical lines are 1 standard error.

For the damage assessments for all trees per plot combined, tree canopy damage indices were not significantly different among the treatments prior to icing (May 2015). In August 2016, the combined mean tree canopy damage indices were significantly greater in the Mid and High treatment plots compared to the Control and Low treatment plots, with the Mid×2 at an intermediate value. In August 2017, the combined mean tree canopy damage indices were significantly greater in the Mid×2 treatment plots compared to the Control plots (Fig 7). In August 2018, the combined mean tree canopy damage indices were significantly greater in the Mid, Mid×2, and High treatment plots compared to the Controls, and by July 2019, the combined mean tree canopy damage indices were significantly higher in all four treatments compared to the Controls (Fig 7).

The combined mean canopy tree damage indices increased in all treatments, including the Control, over the five years of the study, but the Mid, Mid×2 and High treatment plots showed a greater increase in damage over time than the Control and Low treatment plots, which resulted in the significantly higher damage indices for the assessments completed in the summers of 2018 and 2019 (Fig 7).

For individual species, American beech had numerically the lowest initial values for mean canopy tree damage indices (1.2 in the Control treatment to 1.6 in the Mid treatment), followed by yellow birch (1.2 in the Mid×2 treatment to 2.3 in the Mid treatment), red maple (1.3 in the Mid×2 treatment to 2.6 in the Control treatment), and then sugar maple (1.6 in the Mid treatment to 2.6 in the Control treatment) (Fig 7).

For American beech, no significant differences were observed in mean tree canopy damage indices among treatments prior to icing (May 2015) (Fig 7). By August 2019, the mean tree canopy damage index for American beech trees in the control plots had not changed whereas values for the indices for the treated plots had increased by 46 to 74%. Initial mean tree damage indices were more variable for yellow birch, sugar maple and red maple than for American beech. By August 2019, mean tree canopy damage indices for the trees in the Control plots for these three species showed only minor changes (0 to +18%), whereas values in treated plots had changed by +38 to +133% for yellow birch, -3 to +67% for sugar maple and +50 to +140% for red maple. The largest numerical increases in mean tree damage indices for each species were in the most severe treatments, with mean indices increasing by 1 unit in the High treatment for American beech, 2 units in the High treatment for yellow birch, 1.5 units in the High treatment for sugar maple, and 1.7 units in the Mid×2 treatment for red maple. Overall, the percent change in mean tree canopy damage indices over the four years of the study was numerically greatest for red maple (in Mid×2 treatment), > yellow birch > American beech > sugar maple; the increases in absolute values of mean tree canopy damage index were numerically greater for yellow birch > red maple (in Mid×2 treatment) > sugar maple > American beech.

Due to small sample size, few of the species year × treatment comparisons were statistically significant (Table 6). Of the 24 possible year × treatment pair-wise comparisons prior to icing (May 2015), 18 showed no significant pairwise differences, five showed tree canopy damage indices greater in sugar maple (2), red maple (2) and yellow birch (1) than American beech, and one showed tree canopy damage index in red maple greater than yellow birch. By August 2019, of the 24 possible year × treatment pair-wise comparisons prior to icing, 20 showed no significant pairwise differences, three showed tree canopy damage indices greater in sugar maple (2) and red maple (1) than American beech, and one showed tree canopy damage index in red maple greater than yellow birch.

**Table 6:** Total numbers of significant (*P*<0.05) pair-wise comparisons for pre-treatment (2015) and post-treatment (2019) tree canopy damage assessments.

Results	Total number	2015 pre-treatment	2019 Post-treatment
No differences	38	18	20
sugar maple > American beech	4	2	2
red maple > American beech	3	2	1
yellow birch > American beech	1	1	0
red maple > yellow birch	2	1	1
Total	48	24	24

## Discussion

Our methods and results demonstrate the feasibility of (i) creating a gradient of experimental ice storms and (ii) creating experimental ice storms in consecutive years. The treatment levels were chosen to reflect the amount of ice accretion that triggers National Weather Service ice storm warnings from the Mid-Atlantic to the New England and New York states (6.4 and 12.7 mm radial ice accretion), and then to extend this to a more extreme level (19.1 mm radial ice accretion), and to include an extreme frequency (12.7 mm radial ice accretion in two consecutive years). This initial work establishes the framework to evaluate the response to and recovery from this experimental suite of ice storms for decades to come.

### Ice accretion

Radial ice accretion was used as a measure of disturbance intensity. Ice accretion is difficult to characterize and quantify as its buildup varies on surfaces of different materials, diameters, shapes and inclinations [39]. Moreover, measurements in larger storms are dangerous. Pleas for better measurements of ice accretion have been made since the early 1960s [40]. Here, a passive ice collector was deployed to create a standardized, uniform and easily replicated collection surface for ice accretion measurements. Ice accretion measured with calipers produced similar values to measurements of water volume when estimated accretions were < 10 mm, with few icicles visibly present. However, caliper ice measurements underestimated ice accretion compared to the water volume measurements when estimated accretion was > 10 mm and abundant icicle formation was observed. This result highlights the difficulty of measuring ice loading with calipers when icicles are present. Combining both methods probably provided the best metric of icing intensity on these experimental plots. More work is needed to develop simple, low cost measurement protocols for ice accretion to standardize collection surfaces and accretion angles and determine how to account for icicle formation.

### Fine and coarse woody debris inputs

The most immediate ecological impact of ice storms on forests is creation of canopy gaps as twigs, branches, tree tops and eventually entire trees break and snap under the load of the accumulated ice. In a related paper on the canopy impacts of this ice experiment, Fahey et al. [28] showed significant post-treatment declines in leaf area index of 27 to 37%, increases in gap light index of up to 200%, and shifts in the vertical structure of the canopy by the summer of 2017 compared to pre-treatment values. They found that changes in canopy properties were significantly related to both ice accretion as well as the annual total mass of FWD coupled with fall foliar litterfall.

Results reported here complement the work of Fahey et al. [28] by quantifying the magnitude and timing of inputs of the FWD and CWD creating the changes in canopy structure following icing. Although these inputs were generally commensurate with treatments (Figs 5 and 6), it is noteworthy that the mass of FWD was significantly greater in the 2017 Mid×2-yr2 treatment compared to both the 2016 Mid and Mid×2-yr1, despite the same target icing (13 mm). Similarly, the mass of CWD was significantly greater and more decayed in the 2017 Mid×2-yr2 treatment compared to both the 2016 Mid and Mid×2-yr1. We propose five non-exclusive explanations for this result: (i) the first year of mid-level icing did not break or damage all the susceptible twigs and branches in the canopy; (ii) the first year of icing broke branches that remained suspended in the canopy until they fell in response to the second year of icing, (iii) the first year of icing weakened or damaged branches that were then more susceptible to breaking during the second year of icing; (iv) the crown loss following the first year of ice treatment opened up access for greater water penetration/ice buildup on remaining twigs and branches, and reduced internal crown support that otherwise could have prevented the excessive bending and additional breakage and loss of canopy components; and (v) for FWD, the amount of time between icing and litter collection was greater in 2017 (70 days) than 2016 (13 to 37 days), although mean and maximum wind speeds between icing and collections were comparable. In any case, these results demonstrate an interactive effect of ice storm frequency and severity that would not be identified without our experimental approach.

Carbon inputs in FWD and CWD, in general, followed mass inputs, with step-wise increases in input commensurate with icing in 2016 and a pulse of C input in the Mid×2-yr2 treatment plots in 2017. The FWD C inputs following the single events in this ice storm experiment ranged from 18% of the average annual fine litter C inputs at HBEF in the Low treatment to 82% of the average annual inputs in the Mid×2-yr2 treatment (Table 7). The CWD C flux inputs following the single events in this experiment ranged from 16% of the average annual CWD C inputs at HBEF in the Low treatment plots, to approximately equal to the average annual CWD C inputs in the Mid, Midx2-yr1 and Midx2-yr 2 treatment plots, to almost five times greater than the average annual CWD C inputs in the high treatment plots (Table 7). These inputs, especially for the High treatment plots, represent a large transfer of C from the canopy to the soil, and accordingly a large amount of organic debris to be managed by foresters and local municipalities. As woody tissues typically have high C/N ratios and high lignin concentrations [41] these inputs may take years to decades to decompose.

**Table 7:** Comparison of estimated ice accretion, fine litter-C, and coarse woody debris-C for the Hubbard Brook Experimental Forest (HBEF) long term mean annual input, ice storm of 1998, HBEF 2012 pilot ice storm experiment, and current ice storm experiment.

	Estimated Ice Accretion (mm)	Fine Litter	Coarse Woody Debris
		(g C m^-2^)	(g C m^-2^)
Long Term Mean Annual Input at HBEF^1^	---	171	140
Ice Storm 1998 Mid Elevation (570–600 m)^2^	5.9	29.4±3.1	---
Ice Storm 1998 High Elevation (700–830 m)^2^	14.4	31.5 ±3.2	434±93
HBEF Pilot Ice Storm Experiment (560 m)^3^	9.35	142 ± 29	218 ±107
HBEF ISE Low (520 m)^4^	5.70 ± 0.22	31.1 ± 0.2	23.7 ± 10.8
HBEF Mid (520 m)^4^	8.51 ± 1.33	70.3 ± 21.6	138.3 ± 44.1
HBEF Mid×2-yr1 (520 m)^4^	14.62 ± 2.23	67.3 ± 13.5	115.3 ± 68.5
HBEF Mid×2-yr2 (520 m)^4^	13.18 ± 0.10	140.8 ± 18.1	181.7 ± 77.1
HBEF High (520 m)^4^	16.35 ± 1.95	120.9 ± 22.8	672.6 ± 182.2

Values are the mean ± SE.

^1^ FWD and CWD from Fahey et al. [42]. CWD is sum of coarse litterfall (20 g C m^-2^ yr^-1^) and coarse woody debris (120 g C m^-2^ yr^-1^).

^2^1998 ice accretion from Rhoads et al. [8]; CWD from Fahey et al. [42]; FWD from Fahey (unpubl.) calculated from dry matter divided by 2.

^3^ FWD and CWD from Rustad and Campbell (17).

^4^ice accretion from volume measurements.

Experimental inputs of FWD C and CWD C can also be compared to inputs incurred at HBEF during the natural ice storm of 1998 (Table 7). This storm severely impacted parts of the south facing watersheds at the HBEF [5, 23]. The FWD C inputs in the Low treatment plots of this experiment were roughly similar to those recorded at mid elevations at HBEF (31 vs 29 g C/m^2^, respectively) (Table 7) after the 1998 ice storm, where the amounts of ice accretions were similar (5.7 mm ice for experiment; 5.9 mm ice for natural storm). The FWD C inputs in the Mid and High treatment plots were approximately two to four times the amount of FWD C inputs at high elevations at HBEF after the 1998 ice storm where ice amounts were roughly similar (8.5 to 16.4 mm ice for experiment and 9.4 mm ice for natural storm) (Table 7). The CWD C inputs in the experimental plots with roughly equivalent icing to that of the natural ice storm (Midx2-yr1, Midx2-yr2 and High) ranged from 27% to 155% the CWD C recorded after the natural ice storm (Table 7).

This current experiment was designed after a pilot ice storm experiment conducted at HBEF in 2012 at a similar elevation and forest type to this experiment [17]. Fine woody debris C inputs for the Mid and Mid×2-yr1 treatments (which had the same target ice accretion as the pilot study (12.7 mm)) were approximately half of the FWD C inputs of the pilot study; FWD C inputs for the Mid×2-yr2 treatments (which has the same target ice accretion as the pilot study but was applied in a second consecutive year) were approximately equal to the FWD C inputs of the pilot study (Table 7). Coarse woody debris C inputs for the Mid and Mid×2-yr1 treatments (which had the same target ice accretion as the pilot study (12.7 mm)) were 64 and 53 percent of the CWD C inputs of the pilot study, respectively; CWD C inputs for the Mid×2-yr2 treatments (which had the same target ice accretion as the pilot study but was applied in a second consecutive year) were 84 percent of the pilot study (Table 7).

Differences in FWD C and CWD C inputs among the several experimental and the natural icing events at HBEF reflect the many factors that interact to influence forest response to ice storms. These include natural variations in species composition, stand age, tree and stand health, soils, and antecedent or postcedent event weather conditions, as well as experimental artifacts such as the more rapid icing (~4 hours) during the experiments versus the natural event (~3 days).

In addition to C inputs, fine and coarse woody debris play an important role in recycling nutrients in northern forest ecosystems. Nitrogen, P and Ca can all be limiting in these forests [43]. Additions of these elements in woody materials replenish forest floor nutrients and, as they are slowly mineralized over time, stimulate future growth. Magnesium and K are also critical for tree health and to repair wounds following injury or insect infestation [44]. The simulated ice storms in this study resulted in significant inputs of N, P, Ca, Mg and K to these forest plots. The nutrient inputs reported here for the combined CWD and FWD measured in the Control (2016 and 2017) and Low treatment plots in 2016 were less than or equal to the amounts reported by Gosz et al. [45]for annual litter inputs in perennial tissues for a mixed hardwood site at a comparable elevation at Hubbard Brook (Table 8). The nutrient inputs reported here for the Mid, Mid×2-yr1 and High treatment plots in 2016 were 137 to1048% higher than those reported by Gosz et al. (1972); and were 318 to 617% higher for the Mid×2_yr2 treatment plots in 2017. Thus the nutrient transfers in these single simulated storm events were equivalent to up to 10 years of average annual nutrient inputs at this site.

**Table 8:** Combined input of Hubbard Brook ice storm experiment coarse and initial fine woody debris, and historic input of litter in perennial woody tissues reproduced from Gosz et al. [45].

	Mass (g m^-2^)	N (g m^-2^)	P (mg m^-2^)	Ca (g m^-2^)	Mg (mg m^-2^)	K (mg m^-2^)
Gosz 1972	217.40	1.18	90.00	1.28	110.00	2100.00
Control 2016	55.15	0.22	12.07	0.27	23.03	44.52
Control 2017	29.72	0.11	5.60	0.16	10.14	17.88
Low 2016	182.93	0.71	35.02	1.27	101.92	96.79
Low 2017	34.48	0.17	8.10	0.22	21.13	30.27
Mid 2016	459.76	2.30	147.00	3.69	206.28	550.58
Mid 2017	101.82	0.43	22.31	0.63	50.06	93.00
Midx2 2016	380.26	1.99	123.36	2.50	181.56	531.01
Midx2 2017	757.00	4.31	349.43	5.58	349.84	1294.86
High 2016	1660.59	6.96	485.84	9.38	720.60	2200.68
High 2017	76.50	0.34	17.39	0.47	35.07	62.56

### Tree canopy damage

The results from the tree canopy damage assessments demonstrated that tree response to ice-induced crown injury unfolds over a period of years. For all species combined, the indices of canopy damage increased over time, with significant difference between Mid and High treatment plots vs Control and Low treatment plots only emerging after two to three years following icing (Fig 7). Similar trends were evident for the four individual deciduous species which had representation in all the plots. It is still early in the recovery period, and despite the high levels of icing and the increase in tree canopy damage indices, few trees were characterized as class 4 (> 75% damage). Continued monitoring of these trees will be needed to reveal the full extent of the damage over time. These results are consistent with the evolution of disease and mortality following ice storms as observed by Shortle et al. [21] and Deschenes et al. [46]. Delayed, lagged and/or persistent declines in tree health following ice storm-induced crown damage may be due to a combination of C starvation (due to loss of canopy foliage and increased C requirements for wound closure and defense) and secondary attacks by insects, pests and pathogens [21, 47].

Four deciduous species were present in all the treatments and provided limited evidence for species-specific differences in response to icing. For example, red maple and yellow birch trees showed the greatest change in tree canopy damage indices over the four years of the study. In contrast, American beech had the lowest initial tree canopy damage indices for all the species, and showed the smallest change in this index in response to the repeated ice applications. It is notable that these responses are for the overstory American beech which were relatively healthy, with little sign of beech bark disease, a major disease that impacts the health of American beech across its range [48]. Observationally, understory American beech trees were considerably bent over by the weight of the ice, and stayed bowed over for the duration of the study, contributing to the canopy affects described by Fahey et al. [49]. The sugar maple in these stands had the highest initial tree canopy damage indices for all the species, and exhibited only moderate increases in response to the treatments. These observations can be contrasted to those reported by Shortle et al. [21]. In their study of 60–120 year old forest stands across New England impacted by the 1998 ice storm, American beech sustained the most damage, followed by yellow birch and then sugar maple. This discrepancy in the susceptibility of American beech to icing between the two studies may be due to the relative lack of a pre-existing stressor such as beech bark disease, the shorter amount of time between the icing and the sampling (as impacts evolve over time), and the small sample size in this study compared to the more comprehensive review including a greater range of sites impacted by pre-existing conditions, as presented by Shortle et al. [21].

Despite these patterns over time, few significant pair-wise species differences in tree canopy damage emerged among the four deciduous species. This may be due, in part, to the small sample size for most species, or to the fact that not all species were present in all treatments, which precluded additional species in the pairwise comparisons. Notably, sample sizes for the two coniferous species present in the plots—red spruce and balsam fir—were not robust enough for pair-wise comparisons. Coniferous species are known to be more resilient to ice storms than deciduous trees [50]. In order to answer the driving question of which species or age classes of species are more or less susceptible to ice storm damage, more synoptic studies of tree damage, likely following natural icing events, are needed.

### Thresholds

Application of an experimental gradient approach to ice storm damage allowed for the identification of several thresholds for this forest stand. Light icing (i.e., target 6.4 mm ice) caused little twig or branch breakage. Although this amount of icing causes slippery conditions on roads and walkways, it had little impact on FWD and CWD in this forest, as reflected in the relatively low amounts of FWD and CWD in the Low treatment plots, which were not significantly different than the Control plots (Figs 5 and 6). The first thresholds in this forest was observed between 6.4- and 12.7-mm target ice accretions, when noticeable amounts of twigs and small branches were observed to break and fall to the ground. This was reflected in the significantly greater accumulations of FWD in the Mid and Mid×2-yr1 treatment plots compared to the Control plots in 2016, and significantly greater accumulation of FWD in the Mid×2-yr2 treatment plots compared to all other plots in 2017. It also contributed to the small increase in CWD in the Mid and Mid×2-yr1 treatment plots compared to the Control and Low treatment plots in 2016, and to the significant increase in CWD in the Mid×2-yr2 treatment plots compared to all other plots in 2017. A second threshold was observed between 12.7- and 19.1-mm target ice accretion, where whole tree tops bent under the weight of accumulated ice and were beginning to snap at the stem. This observation was reflected in the significant pulse of CWD in the High treatment plots compared to all other treatment plots in 2016. Similar tipping points were reported by Lemon [40] in a synthesis of observations from a suite of ice storms affecting forests in New York State, USA. Note that our experiment was conducted under calm conditions (maximum wind speed 1.8 m/s). Wind accentuates forest canopy ice damage, and the tipping points observed here could occur at lower ice accretions under higher wind conditions.

### Hydrologic and chemical artifacts of treatments

In conducting field-scale experiments, potential methodological artifacts that may confound results must be considered. For icing experiments, an artifact can be collateral impacts on forest hydrologic and chemical budgets from the water used to generate ice treatments. Here, the water source for icing was the nearby Hubbard Brook. The chemistry of this stream, studied for over 50 years [51], is characterized as dilute [52] and thus was a good candidate as a water source. Results showed that the amount of water applied to experimental plots was within the range of a large precipitation event at Hubbard Brook (i.e., 5–16% of mean annual precipitation), and similar in magnitude to the water input in the natural ice storm of 1998, with water input to the Low treatment plots being less than water inputs in the natural storm (74%) and water inputs in the High treatment plots being more (254%) (Table 3). Measured throughfall volume was lower than precipitation and ranged from 35 to 140% of the water input measured for the 1998 ice storm.

Chemical inputs in the treatment were measurable but well below commercial forest fertilization levels [53, 54]. Here, total N inputs (NO_3_^-^+ NH_4_^+^ + DON) for the High treatment represented 7% of the average annual precipitation inputs for the site; total base cations input ranged from 63% (K+) to 300% (Ca^2+^) and 317% (Mg^+^) of the average annual precipitation inputs (Table 3). All nutrient inputs were considerably higher than those in the naturally occurring 1998 ice storm (Table 3), representing a possible experimental artifact that needs to be accounted for in future interpretations of biologic and chemical responses to ice storm treatments.

## Conclusions

Currently, a knowledge gap exists on the short- to long-term ecological consequences of extreme weather disturbances. These types of events are problematic to study because they are heterogeneous in time and space, difficult to predict, and disrupt scientific and social infrastructure when they occur, making access to experimental instruments and research sites challenging. Extreme event experiments can help fill this gap, but they can be difficult and even risky to undertake.

Initial results from this study provide quantitative measurements of FWD and CWD mass and nutrient transfers from forest canopies to soil in response to different intensities and frequencies of canopy ice accretion. These metrics of response can help foresters, emergency managers, town planners, electric power utilities, arborists, winter recreationists and others be better prepared for the aftermath of ice storms. In particular, results suggest that mature northern hardwood forests such as the one studied here will sustain little damage below 6.4 mm radial ice accretion, moderate damage with up to 12.7 mm of accretion, and significant damage to crowns with upwards of 19.1 mm of ice or more. Further, current ice storm warnings that are issued by the National Weather Service in New York and New England for ≥12.7 mm radial ice accretion may be too high because significant FWD and some CWD will likely have already fallen at that level of icing and damage will have been done. The 6.4 mm ice storm warnings as issued for the Mid-Atlantic states may provide a safer level to alert the public to pending tree damage from ice storms. Results also suggest that first responders need to be prepared for an equal or greater amount of organic debris inputs if large storms (here ≥12.7 mm radial ice accretion) occur in back-to-back years. Our results suggest that damaged trees, branches or whole trees that do not fall in the first year are more susceptible to further damage in an ensuing year.

This experiment was the first of its kind in creating a suite of experimental ice storms of different intensities and frequencies in a forest ecosystem. Recommendations for future work include: (i) increase intensity of icing to simulate a tipping point that marks a state change characterized by long-lasting alterations of stand composition or structure; (ii) increase frequency of icing within a single year and over a greater number of consecutive years to better understand how resilient trees are to repeat ice storms and identify tipping points characterized by a state change; (iii) conduct a similar gradient of ice storms in different vegetation types and age classes to better understand what vegetation types and age classes are more or less susceptible to ice damage; (iv) conduct the experiment under different wind speeds, as greater damage is likely with higher wind velocities; and (v) increase plot size to encompass the complete canopies and root systems of a larger number of trees to minimize belowground interactions from adjacent non-iced trees. More work is also recommended to develop simple, low cost measurement protocols for ice accretion, including a refined design for the passive collector that optimizes collector surfaces, angles, and approaches to minimize icicle formation (Campbell et al. in review). Such collectors would allow researchers and citizen scientists to gather more standardized information on the intensity and frequency of icing events across the landscape.

The most interesting results from this experiment are likely those that will evolve over time as this forest ecosystem reorganizes and recovers from this suite of experimental ice storms. This study provides an experimental framework to study the short- and long-term impacts of these experimental ice storms on the structure and function of this northern hardwood forest now [28, 55, 56] and in the decades to come.

## Supporting information

S1 Fig Spraying water to create experimental ice storms at the Hubbard Brook Experimental Forest, NH. (JPG)

S1 Photo license form (PDF)

## References

[1] Wuebbles D, Meehl G, Hayhoe K, Karl TR, Kunkel K, Santer B, et al CMIP5 Climate Model Analyses: Climate Extremes in the United States. Bulletin of the American Meteorological Society. 2014;95(4):571–83. 10.1175/bams-d-12-00172.1

[2] IPCC CC. Synthesis Report.(2014). Contribution of Working Groups I. II and III to the Fifth Assessment Report of the Intergovernmental Panel on Climate Change Core Writing Team In: RK Pachauri and LA Meyer (Eds) IPCC, Geneva, Switzerland. 2014;151.

[3] Arnone JA, Jasoni RL, Lucchesi AJ, Larsen JD, Leger EA, Sherry RA, et al A climatically extreme year has large impacts on C4 species in tallgrass prairie ecosystems but only minor effects on species richness and other plant functional groups. Journal of Ecology. 2011;99(3):678–88. 10.1111/j.1365-2745.2011.01813.x

[4] Jentsch A, Kreyling J, Beierkuhnlein C. A new generation of climate-change experiments: events, not trends. Frontiers in Ecology and the Environment. 2007;5(7):365–74. 10.1890/1540-9295(2007)5[365:Angoce]2.0.Co;2

[5] Houlton BZ, Driscoll CT, Fahey TJ, Likens GE, Groffman PM, Bernhardt ES, et al Nitrogen dynamics in ice storm-damaged forest ecosystems: implications for nitrogen limitation theory. Ecosystems. 2003;6(5):431–43.

[6] Smith MD. An ecological perspective on extreme climatic events: a synthetic definition and framework to guide future research. Journal of Ecology. 2011;99:656–63.

[7] Hufkens K, Friedl MA, Keenan TF, Sonnentag O, Bailey A, O’Keefe J, et al Ecological impacts of a widespread frost event following early spring leaf-out. Global Change Biology. 2012;18(7):2365–77. 10.1111/j.1365-2486.2012.02712.x

[8] Rhoads AG, Hamburg SP, Fahey TJ, Siccama TG, Hane EN, Battles J, et al Effects of an intense ice storm on the structure of a northern hardwood forest. Canadian Journal of Forest Research. 2002;32:1763–75.

[9] Battles JJ, Cleavitt NL, Saah DS, Poling BT, Fahey TJ. Ecological impact of a microburst windstorm in a northern hardwood forest. Canadian Journal of Forest Research. 2017;47(12):1695–701. 10.1139/cjfr-2017-0206

[10] Groffman PM, Rustad LE, Templer PH, Campbell JL, Christenson LM, Lany NK, et al Long-Term Integrated Studies Show Complex and Surprising Effects of Climate Change in the Northern Hardwood Forest. BioScience. 2012;62(12):1056–66. 10.1525/bio.2012.62.12.7

[11] Plotkin AB, Foster D, Carlson J, Magill A. Survivors, not invaders, control forest development following simulated hurricane. Ecology. 2013;94(2):414–23. 10.1890/12-0487.1 23691660

[12] Zimmerman JK, Hogan JA, Shiels AB, Bithorn JE, Carmona SM, Brokaw N. Seven-year responses of trees to experimental hurricane effects in a tropical rainforest, Puerto Rico. Forest Ecology and Management. 2014;332:64–74. 10.1016/j.foreco.2014.02.029.

[13] Kayler ZE, De Boeck HJ, Fatichi S, Grünzweig JM, Merbold L, Beier C, et al Experiments to confront the environmental extremes of climate change. Frontiers in Ecology and the Environment. 2015;13(4):219–25. 10.1890/140174

[14] Changnon S. Characteristics of ice storms in the United States. Journal of Applied Meteorology. 2003;42:630–9.

[15] Sun G, McNulty SG, Moore Myers JA, Cohen EC. Impacts of multiple stresses on water demand and supply across the southeastern United States. Journal of the American Water Resources Association. 2008;44(6):1441–57.

[16] Ding Y, Jia X, Wang Z, Chen X, Chen L. A contrasting study of freezing disasters in January 2008 and in winter of 1954/1955 in China. Frontiers of Earth Science in China. 2009;3(2):129–45. 10.1007/s11707-009-0028-2

[17] Rustad LE, Campbell JL. A novel ice storm manipulation experiment in a northern hardwood forest. Canadian Journal of Forest Research. 2012;42(10):1810–8. 10.1139/x2012-120

[18] Nagel TA, Firm D, Rozenbergar D, Kobal M. Patterns and drivers of ice storm damage in temperate forests of Central Europe. European Journal of Forest Research. 2016;135(3):519–30. 10.1007/s10342-016-0950-2

[19] Cheng CS, Li G, Auld H. Possible Impacts of Climate Change on Freezing Rain Using Downscaled Future Climate Scenarios: Updated for Eastern Canada. Atmosphere-Ocean. 2011;49(1):8–21. 10.1080/07055900.2011.555728

[20] Swaminathan R, Sridharan M, Hayhoe K. A Computational Framework for Modelling and Analyzing Ice Storms. arXiv e-prints [Internet]. 2018 May 01, 2018. https://ui.adsabs.harvard.edu/abs/2018arXiv180504907S.

[21] Shortle WC, Smith KT, Dudzik KR. Tree survival 15 years after the ice storm of January 1998. Res. Pap. NRS-25. Newtown Square, PA: U.S. Department of Agriculture, Forest Service, Northern Research Station, 2014.

[22] Irland LC. Ice storms and forest impacts. The Science of the Total Environment. 2000;262:231–42. 10.1016/s0048-9697(00)00525-8 11087029

[23] Irland LC. Ice storm 1998 and the forest of the northeast. Journal of Forestry. 1998;96:32–40.

[24] Seischab FK, Bernard JM, Eberle MD. Glaze storm damage to western New York forest communities. Bulletin of the Torrey Botanical Club. 1993;120:64–72.

[25] Likens G, Dresser B, Buso D. Short-Term Temperature Response in Forest Floor and Soil to Ice Storm Disturbance in a Northern Hardwood Forest2004. 209–19 p.

[26] Hooper M, Arii K, Lechowicz M. Impact of a major ice storm on an old-growth hardwood forest. Canadian Journal of Botany-revue Canadienne De Botanique—CAN J BOT. 2001;79:70–5.

[27] Weeks BC, Hamburg SP, Vadeboncoeur MA. Ice storm effects on the canopy structure of a northern hardwood forest after 8 years. Canadian Journal of Forest Research. 2009;39:1475–83.

[28] Fahey RT, Atkins JW, Campbell JL, Rustad LE, Duffy M, Driscoll CT, et al Effects of an experimental ice storm on forest canopy structure. Canadian Journal of Forest Research. 2019:136–45. 10.1139/cjfr-2019-0276

[29] Bailey AS, Hornbeck JW, Campbell JC, Eagar C. Hydrometeorological database for Hubbard Brook Experimental Forest: 1955–2000 Gen. Tech. Rep. NE-305. Newtown Square, PA: U.S. Department of Agriculture, Forest Service, Northeastern Research Station 36 p. 2003.

[30] Jones KF, Mulherin ND. An evaluation of the severity of the January 1998 ice storm in northern New England. Hanover, NH: U.S. Army Cold Regions Research and Engineering Laboratory, Snow and Ice Division, 1998.

[31] NWS. National Weather Service Reference Guide. In: Commerce USDo, editor.: National Oceanic and Atmopsheric Administration; 2011. p. 134.

[32] EPA. “Method 3052. Microwave Assisted Digestion of Siliceous and Organically Based Matrices. In: Agency EP, editor. Test Methods for Evaluating Solid Wastes: Physical/Chemical Methods. I. 3rd ed. Washington, D.C.: U.S. Environmental Protection Agency, Office of Solid Waste and Emergency Response; 1996. p. 3052–1–-20.

[33] Waddell KL. Sampling coarse woody debris for multiple attributes in extensive resource inventories. Ecological Indicators. 2002;1(3):139–53. 10.1016/S1470-160X(01)00012-7.

[34] Shortle WC, Smith KT, Dudzik KR. Tree Survival and Growth Following Ice Storm Injury. 2003.

[35] Bates D, Mächler M, Bolker B, Walker S. Fitting Linear Mixed-Effects Models Using lme4. 2015. 2015;67(1):48 Epub 2015-10-07. 10.18637/jss.v067.i01

[36] Team RC. R: A language and environment for statistical computing. 2019.

[37] Lenth R. emmeans: Estimated marginal means, aka least-squares means. 2019.

[38] Christensen RHB. ordinal—Regression Models for Ordinal Data 2019. https://CRAN.R-project.org/package=ordinal.

[39] Campbell JL, Rustad L.E., Garlick S., Newman N., Stanovick J.S., Halm I., et al Development of a low-cost ice accretion measurement methodology for a volunteer observation network. Applied Meteorology and Climatology 2020.

[40] Lemon PC. Forest Ecology of Ice Storms. Bulletin of the Torrey Botanical Club. 1961;88(1):21–9. 10.2307/2482410

[41] Johnson CE, Siccama TG, Denny EG, Koppers MM, Vogt DJ. In situ decomposition of northern hardwood tree boles: decay rates and nutrient dynamics in wood and bark. Canadian Journal of Forest Research. 2014;44(12):1515–24. 10.1139/cjfr-2014-0221

[42] Fahey TJ, Siccama TG, Driscoll CT, Likens GE, Campbell J, Johnson CE, et al The biogeochemistry of carbon at Hubbard Brook. Biogeochemistry. 2005;75:109–79.

[43] Vadeboncoeur MA. Meta-analysis of fertilization experiments indicates multiple limiting nutrients in northeastern deciduous forests. Canadian Journal of Forest Research. 2010;40(9):1766–80. 10.1139/X10-127

[44] Boxman AW, Cobben PLW, Roelofs JGM. Does (K+Mg+Ca+P) fertilization lead to recovery of tree health in a nitrogen stressed Quercus rubra L. stand? Environmental Pollution. 1994;85(3):297–303. 10.1016/0269-7491(94)90051-5 15091660

[45] Gosz JR, Likens GE, Bormann FH. Nutrient content of litter fall on the Hubbard Brook experimental forest, New Hampshire. Ecology. 1972;53(5):769–84.

[46] Deschênes É, Brice M-H, Brisson J. Long-term impact of a major ice storm on tree mortality in an old-growth forest. Forest Ecology and Management. 2019;448:386–94. 10.1016/j.foreco.2019.06.018.

[47] Smith KT, Shortle WC. Radial growth of hardwoods following the 1998 ice storm in New Hampshire and Maine. Canadian Journal of Forest Research. 2003;33:325–9.

[48] Leak WB. Fifty-year impacts of the beech bark disease in the Bartlett Experimental Forest, New Hampshire. Northern Journal of Applied Forestry. 2006;23(2):141–3.

[49] Fahey RT, Atkins JW, Campbell JL, Rustad LE, Duffy M, Driscoll CT, et al Effects of an experimental ice storm on forest canopy structure. Canadian Journal of Forest Research. 2019;50:136–45. 10.1139/cjfr-2019-0276

[50] Carvell TE K L, True RP. Effects of glaze on the development of Appalachian hardwoods. Journal of Forestry. 1957;55:130–2.

[51] Likens GE. Fifty years of continuous precipitation and stream chemistry data from the Hubbard Brook ecosystem study (1963–2013). Ecology. 2017;98(8):2224-. 10.1002/ecy.1894 28763582

[52] Likens GE, Buso DC. Dilution and the elusive baseline. Environmental Science & Technology. 2012;46(8):4382–7. 10.1021/es3000189 22455659

[53] Sullivan TP, Sullivan DS. Influence of nitrogen fertilization on abundance and diversity of plants and animals in temperate and boreal forests. Environmental Reviews. 2017;26(1):26–42. 10.1139/er-2017-0026

[54] Turkington R, John E, Krebs CJ, Dale MRT, Nams VO, Boonstra R, et al The effects of NPK fertilization for nine years on boreal forest vegetation in northwestern Canada. Journal of Vegetation Science. 1998;9(3):333–46. 10.2307/3237098

[55] Weitzman JN, Groffman PM, Campbell JL, Driscoll CT, Fahey RT, Fahey TJ, et al Ecosystem Nitrogen Response to a Simulated Ice Storm in a Northern Hardwood Forest. Ecosystems. 2019 10.1007/s10021-019-00463-w

[56] Yang Y, Meng L, Yanai RD, Montesdeoca M, Templer PH, Asbjornsen H, et al Climate change may alter mercury fluxes in northern hardwood forests. Biogeochemistry. 2019;146(1):1–16. 10.1007/s10533-019-00605-1

