# Supplementary figures and images for: Experimental approach and initial forest response to a simulated ice storm experiment in a northern hardwood forest
Source: PLoS One. 2020 Sep 25;15(9):e0239619. doi: 10.1371/journal.pone.0239619 (PMC7518631; doi:10.1371/journal.pone.0239619)

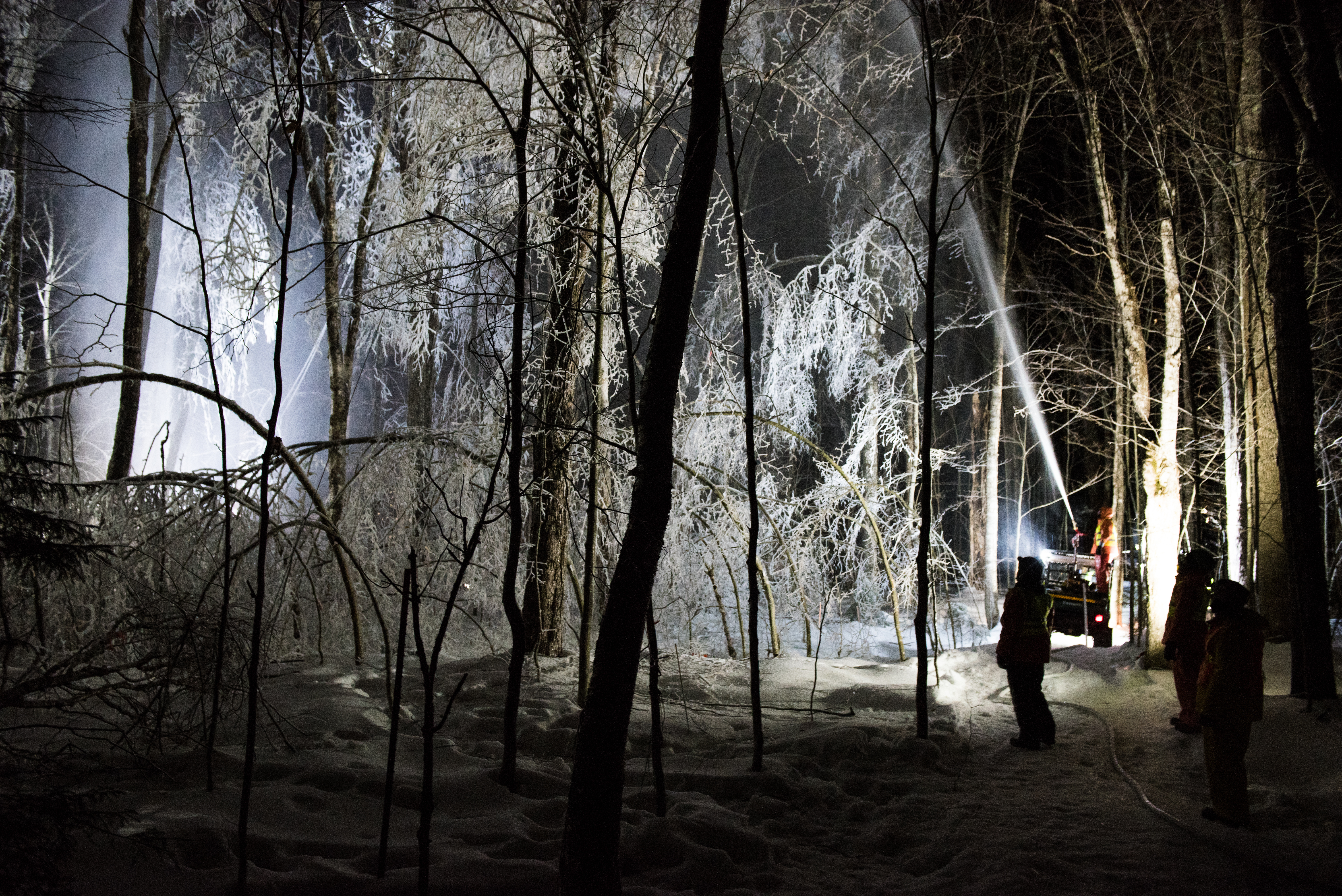

Supplement: S1 Fig — (JPG) [file pone.0239619.s001.jpg]
