# Supplementary material for: Experimental approach and initial forest response to a simulated ice storm experiment in a northern hardwood forest
Source: PLoS One. 2020 Sep 25;15(9):e0239619. doi: 10.1371/journal.pone.0239619 (PMC7518631; doi:10.1371/journal.pone.0239619)
Supplement: S1 Photo license form — (PDF) [file pone.0239619.s002.pdf]

Request for Permission to Publish Content under CC-BY License

Dear Rights Holder or Representative,

I have submitted a paper for publication in a PLOS journal, and wish to include the content listed below in the paper. I'm hereby requesting your (or your company's or institution's) permission to include the content in my paper. Please note that all PLOS journals are published under a Creative Commons Attribution License (CC BY), which allows for unrestricted use and distribution, even commercial, as long as attribution is given to the creator or rights holder of the content. See https://creativecommons.org/licenses/by/4.0/.

To grant me permission to use the content in my PLOS paper, please fill in the information below and then scan the completed form and send it to me at my email address.

Thank you.

My name:

Sarah Garlick

My email address:

sgarlick@hubbardbrookfoundation.org

Description of the content which I'm seeking permission to use (citation and/or title, and pasted screen shot, if applicable):

S1 photo.jpg:

Photograph of the Ice Storm Experiment at the Hubbard Brook Experimental Forest. Photograph by Joe Klementovich, courtesy of the Hubbard Brook Research Foundation.

Link to the Content:

* * *

On behalf of myself or the rights holder, I hereby grant the permission sought herein.

Signature of Party Granting Permission:

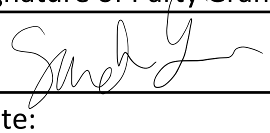

Date:

June 12, 2020

Printed Name and Title:

Sarah Garlick
Director of Science Policy and Outreach
Hubbard Brook Research Foundation
